# Development of aqueous-based multi-herbal combination using principal component analysis and its functional significance in HepG2 cells

**DOI:** 10.1186/s12906-019-2432-9

**Published:** 2019-01-15

**Authors:** Pardeep Kaur, Robin, Rajendra G. Mehta, Balbir Singh, Saroj Arora

**Affiliations:** 1 Department of Botanical & Environmental Sciences, Guru Nanak Dev University, Amritsar, Punjab 143005 India; 2 Cancer Biology Division, IIT Research Institute and Department of Biological Sciences, Illinois Institute of Technology, Chicago, IL 60616 USA; 3 Department of Pharmaceutical Sciences, Guru Nanak Dev University, Amritsar, 143005 India

**Keywords:** Medicinal plants, Antioxidant, HPLC, Principal component analysis, Multi-herbal combination, HepG2

## Abstract

**Background:**

The present study was carried out to prepare multi-herbal combination via comparing antioxidant activity and polyphenolic composition of five medicinal plant extracts of *Punica granatum* L., *Putranjiva roxburghii* Wall., *Swertia chirata* Buch.-Ham., *Tinospora cordifolia* (Willd.) Miers and *Trigonella corniculata* L.

**Methods:**

The herbs were individually evaluated using in vitro antioxidant assays and analyzed by HPLC-PDA. The resultant data was examined using principal component analysis (PCA). Further, herbal combination was prepared on the basis of PCA.

**Results:**

The PCA divided the plants into three groups. The leading or primary group contained *P. granatum* and *P. roxburghii* with the highest antioxidant activity strongly correlated with high amount of kaempferol. *S. chirata* was acknowledged as nourisher herb in one and *T. cordifolia* and *T. corniculata* were identified as stimulator herbs in other group. The herbal combination exhibited high antioxidant activity as compared to the individual plants. The combination revealed good antiproliferative efficacy against hepatocellular carcinoma (HepG2) cells with IC_50_ of 75.864 μg/ml.

**Conclusions:**

The activity observed in vitro with HepG2 cells suggests that the herbal combination can provide therapeutic activity in vivo in future. The study may provide information regarding precise preparation of multi-herbal formulations using PCA as a tool in pharmaceutical industries.

**Electronic supplementary material:**

The online version of this article (10.1186/s12906-019-2432-9) contains supplementary material, which is available to authorized users.

## Background

Phytotherapeutic management of pathological conditions encompasses combinatorial intervention of multiple bioactive constituents manifesting multi-target strategy [1]. The polyphenols from these strategies represent an important diverse cluster of phytochemicals primarily contributing to the remedial measures and prevention of many diseases. These phytoconstituents are usually hydrophobic in nature and their hydrophobicity is intermediate between vitamin C (highly hydrophilic) and vitamin E (highly hydrophobic) [2]. Therefore, their extraction primarily includes solvents like acetonitrile, methanol, acetone and ethanol or their non-aqueous and aqueous admixtures. Thus, contemporary research has been mainly driven towards the use of these solvents neglecting their adverse impact on environment and biology [3]. In addition, the removal of solvent residues requires additional laborious purification steps. There is a major concern regarding the delivery of the effective dose of these polyphenols to target organs due to their lower absorptive tendency in colon [4]. The bioavailability of most of these constituents depends on their water solubility and metabolism in body. The clinical success of these components as a drug has been a limiting factor due to their poor solubility in water, fast metabolic rate or both [5]. Hence, naturally developed water based multi-herbal formulations may circumvent the shortcomings of poor bioavailability without compromising their medicinal value. Water at elevated temperature under pressure is comparable to organic solvent extraction [6]. The elevated temperature reduces the dielectric constant of water making it competent to dissolve even hydrophobic organics such as polycyclic aromatic hydrocarbons and polychlorinated biphenyl analogs. However, the role of pressure is to maintain the liquid state of aqueous mixture and has no significant effect on the dielectric constant of water [6]. In addition very few studies have focussed on cancer preventive or therapeutic agents extracted with water from the plants. In this report we have examined the activity of aqueous extracts of several selected herbs both alone as well as in combination.

The selection of plants considered in this treatise is based on their traditional uses and commercial significance. Five aqueous herbal extracts from *Punica granatum* L., *Putranjiva roxburghii* Wall., *Swertia chirata* Buch.-Ham., *Tinospora cordifolia* (Willd.) Miers and *Trigonella corniculata* L. were used in the present study. A comparative investigation was carried out to portray the antioxidant prospective of water extracts of these five plants belonging to different families. In order to get a more extensive depiction, we examined the antioxidant capacities and polyphenolic composition(s) of different eco-solvent extracted medicinal plant extracts supported by the HPLC-PDA analysis. To the best of our knowledge there is a no report describing specific way for the preparation of multi-herbal formulations. The properties of different herbs in multi-herbal formulations encompass three vital points. First is to identify the primary herb(s), second is the recognition of nourisher herb(s) and third is categorizing active stimulator herb(s). Subsequently, in order to systematically generate multi-herbal combination, the multidimensional variables (antioxidant activities and componential profiles) were statistically evaluated. We performed principal component analysis (PCA) to compare the antioxidative capability of five medicinal plants. The newly identified herbal combination was further evaluated for antiproliferative activity against human malignant cancer cell lines. Thus, the study highlights relevant comprehension with respect to the selection of perceptive combination of herbal plants, for preparing multi-herbal formulations using PCA and reducing the laborious efforts of ‘hit and miss’ methods in pharmaceutical industries.

## Methods

### Chemicals and reagents

DPPH (2–2’diphenyl-1-picrylhydrazyl), gallic acid, catechin, chlorogenic acid, epicatechin, caffeic acid, umbelliferone, coumaric acid, rutin, ellagic acid, quercetin, and kaempferol with ≥90% purity were acquired from Sigma-Aldrich, Bangalore (India). HPLC grade methanol and water were used for HPLC analysis. All other reagents and chemicals used in the present investigation were of analytical grade.

### Procurement of herbal raw materials

The preferred plant parts were from different families (Table 1). The well-authenticated and validated dried samples of *Punica granatum* L. (peel), *Swertia chirata* Buch.-Ham. (whole herb), *Tinospora cordifolia* (Willd.) Miers (stem) and *Trigonella corniculata* L. (seeds) were procured from Herbal Health Research Consortium (HHRC) Pvt. Ltd. Amritsar (India), a reputed government approved Ayurvedic, Siddha and Unani Drug Testing Laboratory. The leaves of *Putranjiva roxburghii* Wall. were collected from the Botanical Garden, Guru Nanak Dev University, Amritsar (India). Plant samples of *P. granatum*, *P. roxburghii*, *S. chirata*, *T. cordifolia* and *T. corniculata* were authenticated and verified by Mr. Viney (Research Officer), Herbal Health Research Consortium (HHRC) Pvt. Ltd. Amritsar (India) by observing the characteristic anatomical features with pharmacognostic studies. All samples were deposited in the herbarium of HHRC Pvt. Ltd. Amritsar (India) with voucher numbers ANC-04, PUT-02, CRT-09, GIL-46 and MET-10 respectively.

**Table 1:** Details of five plant parts used with botanical and vernacular names

S. No.	Botanical name	Hindi name	Part used	Family
1.	*Punica granatum* L.	Anar	Peel/Rind	Punicaceae
2.	*Putranjiva roxburghii* Wall.	Putrajeevak	Leaves	Putranjivaceae
3.	*Swertia chirata* Buch.-Ham.	Chirayata nepali	Whole plant	Gentianaceae
4.	*Tinospora cordifolia* (Willd.) Miers	Giloy	Stem	Menispermaceae
5.	*Trigonella corniculata* L.	Kasuri methi	Seeds	Fabaceae

### Preparation of herbal extracts and multi-herbal combination

The disintegrated samples (100 g each) were soaked separately in stainless steel vessels containing potable water for overnight in the ratio 1:16 (*w*/*v*) at room temperature under aseptic conditions. Then the samples were heated at boiling temperature on a mild flame to reduce it to one quarter of original volume [7, 8]. The decoctions were allowed to cool and filtered using five layered muslin cloth followed by Whatman filter sheet no. 1. Whereas, for herbal combination, the disintegrated samples (in calculated proportion of 33, 27, 25, 10 and 5% for *P. granatum*, *P. roxburghii, S. chirata*, *T. cordifolia* and *T. corniculata* respectively) were soaked together and decoction was prepared as described above for individual plants. Principal component analysis was employed for the calculation of percentage proportion of plants used in the preparation of multi-herbal combination as explained later in the results section. The filtered decoctions were then evaporated to dryness on water bath and extracts were stored at 4 °C till further use.

### Antioxidant analysis

#### Total antioxidant assay

The methodology proposed by Gul et al. [9] was followed for determining the propensity of herbal extracts to reduce molybdate ions. The decocted extracts of each plant (1000 μg/ml) were added to the reagent mixture consisting sulphuric acid (0.6 M), sodium phosphate (28 mM) and ammonium molybdate (4 mM). The incubation of the reaction mixture was carried out at 95 °C for 90 min. After cooling at room temperature, the absorbance was measured at 695 nm using ELISA microplate reader (Synergy HT, BioTek). The ascorbic acid was selected to obtain a standard curve with a regression equation y = 0.002x-0.050 and total antioxidant potential was expressed in terms of ascorbic acid equivalents (AAE).

#### 2–2’Diphenyl-1-picrylhydrazyl (DPPH) free radical scavenging assay

The scavenging activity of herbal extracts was investigated by following the procedure reported by Kang et al. [10] using 2–2’diphenyl-1-picrylhydrazyl (DPPH) radicals as substrate. The different concentrations of extracts were added to DPPH solution (0.1 mM) and incubated for 30 min. The decrease in the absorbance was monitored at 517 nm using ELISA microplate reader (Synergy HT, BioTek). Gallic acid was employed as a positive reference. The scavenging of 2–2’diphenyl-1-picrylhydrazyl radicals by different extracts was expressed as percentage inhibition using Eq 1.

#### Ferricyanide ion reduction assay

The reduction potential of different extracts to reduce ferricyanide ions to ferrocyanide ions was interpreted by the method described by Kalyana Sundaram et al. [11]. The assay mixture contained different concentrations of extracts with phosphate buffer (200 mM, pH 6.6) and potassium ferricyanide (1%). This reaction mixture was kept for 20 min at 50 °C after which trichloroacetic acid (10%) was added to the mixture and centrifugation was carried out at 3000 rpm for 10 min. The supernatant thus obtained was mixed with distilled water and ferric chloride (0.1%) and color developed was measured at 700 nm using ELISA microplate reader (Synergy HT, BioTek). The comparison of the results was done using gallic acid as a positive control with a regression equation y = 16.6ln(x)-15.7. The percentage reduction was calculated using the Eq 2.

#### Lipid peroxidation assay

The defensive effect of decocted extracts was analyzed according to the methodology given by Kumar and Pandey with slight modifications [12]. The different concentrations of extracts were mixed with KCl (0.15 M) and egg yolk homogenate (10% *w*/*v*). Ferric chloride (10 mM) was used to initiate the peroxidation following incubation at 37 °C for 30 min. Formation of thiobarbituric acid reactive substances (TBARS) indicates extent of lipid peroxidation. Therefore, the estimation of TBARS was carried out by addition of ice-cold HCl (0.25 N) containing trichloroacetic acid (15%), thiobarbituric acid (0.5%) and butylated hydroxytoluene (0.5%) to the assay mixture. The mixture was then heated at 100 °C for 60 min. After cooling and centrifugation, absorbance of the supernatants was read at 532 nm using ELISA microplate reader (Synergy HT, BioTek). The antioxidant activity was evaluated in percentage by using Eq 1.

#### Cupric ion reduction assay

The cupric ion reducing antioxidant capacity (CUPRAC) method given by Apak et al. [13] was performed. The reaction mixture consisted of copper (II) chloride (10 mM), neocuproine (7.5 mM) and ammonium acetate buffer (1.0 M) with different concentrations of extracts followed by addition of distilled water. The reaction mixture was kept at room temperature for 30 min and ELISA microplate reader (Synergy HT, BioTek) was used to measure the absorbance at 450 nm. Gallic acid was used as a reference to draw the standard curve with regression equation y = 20.3ln(x)-31.1 and antioxidant activity was calculated using Eq 2.

#### Chelation potential for Fe^2+^ ions

The chelating potential of herbal extracts for ferrous ions was assessed by slightly modifying the method given by Navanesan et al. [14]. The different concentrations of extracts prepared in distilled water were mixed with ferrous chloride (2 mM) and the reaction was initiated by the addition of ferrozine (5 mM). The mixture was incubated at room temperature (25 ± 2 °C) for 10 min and the absorbance was observed at 562 nm using ELISA microplate reader (Synergy HT, BioTek). The decrease in the absorbance of herbal extracts was considered as the measure of chelation power. The chelation potential of EDTA (ethylenediaminetetraacetic acid) was also monitored for comparison and the activity was calculated by means of Eq 1.

Formulae used for antioxidant analysis in previous sections:

*For DPPH, Lipid peroxidation and Chelation potential assays, the percentage activity was calculated using the* Eq 1*:*

$$ Activity\ \left(\%\right)=\frac{Absorbance\ of\ control- Absorbance\ of\ sample}{Absorbance\ of\ control}\times 100 $$

*Whereas, for Ferricyanide ion reduction and Cupric ion reduction assays, the percentage activity was calculated using the* Eq 2*:*

$$ Activity\ \left(\%\right)=1-\left(\frac{Absorbance\ of\ control- Absorbance\ of\ sample}{Absorbance\ of\ control}\right)\times 100 $$

#### Cell-based antioxidant protection in erythrocytes (CAP-e) assay

CAP-e assay was used to evaluate the antioxidant capacity of five plant parts and prepared herbal combination as per the method described by Honzel et al. [15]. This cellular assay utilizes red blood cells as a model to evaluate the protection afforded by antioxidant. In this procedure, whole blood (B^+^) was collected from healthy donors. The plasma, peripheral blood mononuclear cells, and polymorphonuclear cells were removed and the remaining packed red blood cell (RBC) fraction was washed three times in 10–14 ml phosphate buffered saline (PBS) by centrifugation at 2400 rpm for 10 min. After the completion of third washing, the supernatant was removed and 200 μl packed red blood cells were pipetted into 10 ml PBS. The red blood cell aliquots were stored at 4–8 °C until use. Then, concentrations of the extract and the standard gallic acid were prepared and pre-incubated with 100 μl of the RBC suspension for 20 min at room temperature in the dark. This step was followed by three times washing of red blood cells with PBS by centrifugation for 2 min at 2400 rpm to remove any unabsorbed antioxidants. 70 μl of the red blood cells were transferred to a black microplate and 50 μl of 2′-7′dichlorofluorescein diacetate (DCF-DA) was added to each well. The stock solution of fluorescent probe was prepared by mixing 50 μg DCF-DA in 0.2 ml dimethyl sulfoxide and working solution was prepared by adding 0.01 ml of stock to 10 ml of PBS. The microplate was incubated for 20 min and thereafter, red blood cells were exposed to 50 μl H_2_O_2_ (167 mM) for 45 min to induce oxidative stress. Then, the fluorescence intensity was recorded with excitation at 485 nm and emission at 535 nm. The red blood cells were taken as negative control while red blood cells exposed to an oxidative challenge were considered as positive controls.

$$ Inhibition\ \left(\%\right)=\frac{Maximum\ Fluorescence- Fluorescence\ of\ sample}{Maximum\ Fluorescence- Fluorescence\ of\ untreated\ cells}\times 100 $$

### Phenolic and flavonoid content

Total phenolic content of individual plant extract and combination was estimated following the protocol reported by Gupta and Gupta [16]. It consisted of mixing an aliquot of each extract with 1 N Folin-Ciocalteu reagent and 20% sodium carbonate. This mixture was incubated for 2 h and then absorbance was measured at 765 nm. Gallic acid was used as the positive control and the amount of total phenolic content was calculated using regression equation of gallic acid given by y = 0.00181x with a correlation coefficient, *r* = 0.9999. The concentration of phenolic compounds in herbal extracts was expressed as mg gallic acid equivalents (GAE)/100 mg dry weight of extract.

Total flavonoid content of each herbal extract was measured adopting the methodology described by Chanthasri et al. [17]. Briefly, an aliquot of extract was thoroughly combined with distilled water, 5% sodium nitrite and 10% aluminium trichloride solution. After incubation of reaction mixture for 5 min at ambient temperature, 1 M sodium hydroxide was added. The mixture was vortexed and absorbance was recorded at 510 nm using ELISA microplate reader (Synergy HT, BioTek). The total flavonoid content was calculated from the regression equation y = 0.00039x (*r* = 0.9997) obtained by plotting calibration curve of standard catechin and expressed as mg catechin equivalents (CE)/100 mg dry weight of extract.

### High performance liquid chromatography-photo diode array (HPLC-PDA) analysis

#### Preparation of the standard and sample

Standard stock solutions of gallic acid, catechin, epicatechin and rutin were prepared separately at 8 mg/ml (8000 ppm) concentration in HPLC grade methanol. Simultaneously, stock solutions of chlorogenic acid, caffeic acid, umbelliferone, coumaric acid, ellagic acid, quercetin and kaempferol were prepared separately at 4 mg/ml concentration in HPLC grade methanol. Subsequently, each standard solution was mixed in equal proportion to obtain standard mix solution. Furthermore, standard mix solution was 2-fold serially diluted using HPLC grade methanol. The plant extracts were also prepared by dissolving 1 mg of each plant extract in 1 ml of HPLC grade methanol.

#### Apparatus and chromatographic conditions

The analysis was performed on Nexera UHPLC (Shimadzu, Kyoto, Japan). The chromatographic separation and detection was completed using Enable C-18 G (150 × 4.6 mm, 5 μm particle size) column at 25 °C and photo diode array (PDA) detector at 280 nm detection wavelength. The low pressure gradient elution system, using mobile phase, 0.1% acetic acid in water (A) and methanol (B), was used for the separation of peaks with flow rate of 1 ml/min and the 5 μl injection volume. The gradient consists of 70% A and 30% B at start, reaching 45% B at 12 min, up to 75% B at 13.5 min, maintaining 75% B until 15 min, then 50% B at 16.6 min, up to 25% B at 18 min, upholding 25% B until 20 min and re-equilibration of 30% B at 21 min until stopped at 22 min with elution of 4 min.

#### Calibration curves

The mixture of standards for calibration curve was prepared by diluting the stock solution with methanol at 7 concentrations achieved by successive 2-fold serial dilution for quantitative analysis. The calibration curves were constructed by plotting the peak areas versus the concentration of each analyte.

#### Selectivity

The method selectivity was performed by analysis of standard compounds and samples. The peaks of gallic acid, catechin, epicatechin, chlorogenic acid, caffeic acid, umbelliferone, coumaric acid, ellagic acid, rutin, quercetin and kaempferol were identified by comparing their retention times and spectrum with those of the standard peak and spectra.

#### Limit of detection and quantification

The aliquots of the standard solutions (diluted with methanol) were injected at various concentrations. The detection and quantification limit for all the detected standard analytes were calculated on the basis of signal-to-noise ratio (S/N) of 3 and 10.

#### Precision and accuracy

The precision and accuracy of the HPLC method was evaluated for three days separately for intraday and collectively for interday with nine replicates each day. The precision was expressed as percentage relative standard deviation (RSD) and accuracy was calculated as the percentage of recovered amount of polyphenols weighed against standard concentration.

$$ RSD\ \left(\%\right)=\left( standard\ deviation/ mean\ peak\ area\right)\times 100 $$

$$ Recovery\ \left(\%\right)=\left( detected\ concentration/ nominal\ concentration\right)\times 100 $$

### Assessment of antiproliferative potential

#### Measurement of cell proliferation by resazurin cell viability assay

The growth inhibition efficacy of the prepared herbal combination was scrutinized by resazurin cell viability assay following the methodology proposed by Riss et al. [18]. Five human cancer derived cell lines (MG-63, A-549, HeLa, HepG2 and IMR-32) and a non-malignant 293 T cell line were acquired from National Centre for Cell Science (NCCS), Pune (India) (Table 2). The cells were maintained in DMEM or RPMI-1640 growth medium and cells were harvested upon attaining 80–90% confluency followed by centrifugation for 5 min at 1000 rpm at 2–5 °C. The resultant cell pellet was resuspended in complete growth medium to get 1–2 × 10^5^ cells/ml via counting with hemocytometer. The cell suspension (100 μl) was seeded in each well of 96-well tissue culture plate followed by incubation for 24 h in CO_2_ incubator at 37 °C, 5% CO_2_ with 90% relative humidity. The cells attached to the wells were treated with the concentrations of herbal combination ranging from 31.25 to 1000 μg/ml. After 24 h, 20 μl of deep blue colored resazurin solution (freshly prepared) was added to each microplate well and further incubated for 4 h. Then, the fluorescence was recorded at excitation/emission wavelength of 560/590 nm employing ELISA plate reader (Synergy HT, BioTek). Camptothecin (10 μM) was employed as a positive standard and the wells containing medium were considered as negative control in the experiment.

**Table 2:** Human cell lines utilized for the present study

S. No.	Cells	Tissue origin	Malignancy	Cell doubling time
1.	A-549	Lung	Carcinoma	~ 22 h
2.	HeLa	Cervix	Adenocarcinoma	~ 25 h
3.	IMR-32	Sympathetic nervous system	Neuroblastoma	~ 20 h
4.	HepG2	Liver	Hepatocellular carcinoma	~ 48 h
5.	MG-63	Bone	Osteosarcoma	~ 28 h
6.	293 T	Embryonic kidney	Non-malignant	~ 27 h

#### Microscopic visualization of morphological changes in cell and nucleus

For morphological changes in treated and untreated cells, phase contrast microscopy was used. Confocal microscopy was used according to the methodology given by Alam et al. [19] with slight modifications for determination of nuclear changes. HepG2 cells were seeded in a 6 well plate at a concentration of 5 × 10^5^ cells per well for 24 h. Thereafter, the HepG2 cells were treated with IC_50_ of herbal combination and the positive control, camptothecin. Following incubation for 24 h, the cells were washed thrice with phosphate buffered saline (0.1 M) and fixed with paraformaldehye (4%) for 30 min. Finally, the slides were prepared after staining the cells with 4′,6-diamidino-2-phenylindole (DAPI) (10 μg/ml) and Nikon A1R laser scanning confocal microscope system (Nikon Corp., Japan) fitted with Nikon 40X 0.95 NAD-ICM/N2 plan objectives was used to capture images. The acquisition of images and analysis was carried out using the built-in Nikon NIS Element AR software and the fluorescence was observed with a long-pass 488 emission filter.

#### Detection of reactive oxygen species generation

The protocol of Shin et al. [20] was utilized to examine the generation of reactive oxygen species employing HepG2 cell line. These cells were seeded at the density of 3 × 10^5^ cells/well in 1 ml/well growth medium in 24-well tissue culture plate. After 24 h, the cells were treated with IC_50_ concentration of the herbal combination. The treated cells were stained with 10 μg/ml of 2′-7′dichlorofluorescein diacetate (DCF-DA). Then, fluorescence was measured using ELISA plate reader (Synergy HT, BioTek) with excitation and emission wavelengths of 485/20 and 528/20 nm respectively.

#### Assessment of mitochondrial membrane potential

The assessment of alterations in membrane integrity of HepG2 cells was determined using the procedure described by Deng et al. [21]. Following the overnight culture of HepG2 cells (3 × 10^5^ cells/well in 1 ml/well growth medium in 24-well culture plate), the cells were treated with IC_50_ concentration of herbal combination. This treatment was followed by addition of rhodamine 123 dye (10 μg/ml) for an hour. Cells were then washed three times with phosphate buffered saline and the fluorescence was examined using ELISA plate reader (Synergy HT, BioTek) with excitation of 485/20 nm and emission of 528/20 nm.

### Statistical analysis

Data were expressed as mean ± standard error (SE) of three replicate determinations. One way analysis of variance (ANOVA) was used to determine the differences among the means and *p* values ≤0.05 were considered as significant. Principal Component Analysis (PCA) was applied on the HPLC and antioxidant resultant variables. Paleontological Statistics (PAST) software version 3.15 was used for data analysis [22].

## Results

### Antioxidative analysis

#### Reduction of molybdate ions

The ability of decocted extracts to reduce molybdate ions was analyzed by the appearance of green colored complex. The increase in the absorbance of ascorbic acid with the concentration was noted and the regression equation y = 0.002x-0.050 with R^2^ = 0.996 was obtained to interpret the antioxidant capacity of different plant extracts. In view of these results, *P. roxburghii* leaves exhibited the highest potential to reduce the molybdate ions (391.750 ± 0.022 μg AAE/mg of dry extract) followed by peel of *P. granatum*, *S. chirata* whole herb, *T. cordifolia* stem and *T. corniculata* seeds with reduction potential of 372.875 ± 0.013, 184.250 ± 0.010, 155.375 ± 0.015 and 143.375 ± 0.015 μg AAE/mg of dry extract respectively. The order of the molybdate ion reduction capability of different herbal samples was: *P. roxburghii* > *P. granatum* > *S. chirata* > *T. cordifolia* > *T. corniculata*. Further, the herbal combination represented even high propensity to reduce the molybdate ions exhibiting total antioxidant capacity of 445.25 μg AAE/mg of dry extract.

#### Scavenging ability against 2–2’diphenyl-1-picrylhydrazyl (DPPH) free radicals

**Fig. 1:**
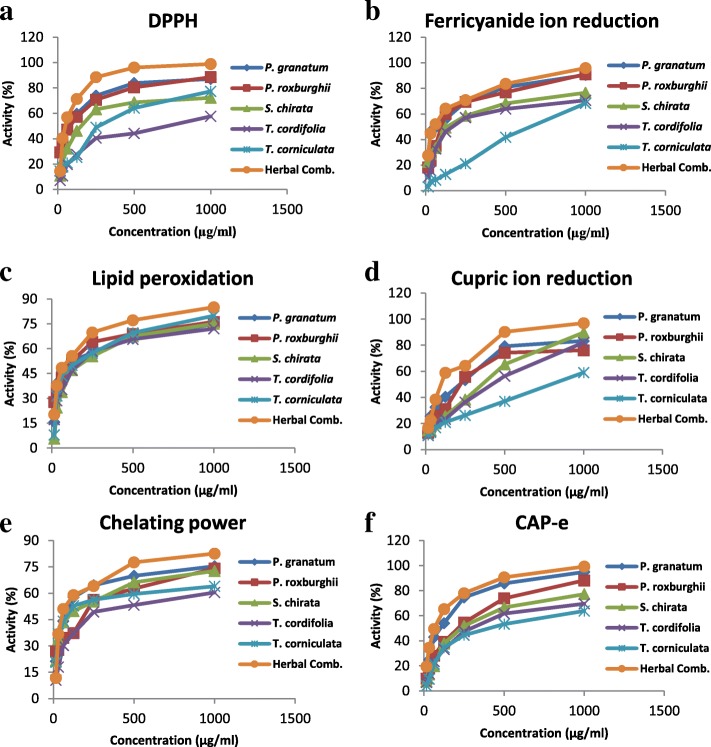
In vitro antioxidant activity of five herbs and herbal combination

The inhibitory action of all the five extracts against 2–2’diphenyl-1-picrylhydrazyl radicals is illustrated in Fig. 1a. The plant extracts exhibited concentration dependent loss of violet color with elevation in percentage scavenging of DPPH radicals. *P. granatum* peel extract recorded the lowest IC_50_ with 64.199 μg/ml concentration indicating good radical scavenging action whereas *T. cordifolia* stem extract showed the least scavenging property among five extracts with highest IC_50_ of 651.780 μg/ml. The order of ranking based on IC_50_ values with respect to scavenging of DPPH radicals was: *P. granatum* < *P. roxburghii* < *S. chirata* < *T. corniculata* < *T. cordifolia.* As compared to the antioxidant ability of individual plant extracts, the herbal combination presented 98.88% reduction of DPPH radicals at the highest tested concentration (1000 μg/ml) with the lowest IC_50_ of 55.433 μg/ml. Additional file 1: Table S1.

#### Reduction of ferricyanide ions to ferrocyanide ions

The reducing power of plant extracts signifying breakage of free radical chain was analyzed by their reduction ability to reduce ferricyanide ions to ferrocyanide ions (Fig. 1b). *P. granatum* peel extract demonstrated the lowest 50% inhibitory concentration of 63.432 μg/ml in comparison to *P. roxburghii, S. chirata, T. cordifolia, T. corniculata*. The lowest IC_50_ of *P. granatum* highlighted it to be the better reductant of ferricyanide ions among five extracts and *T. corniculata* was emerged to possess least reduction potential. Considering the IC_50_ values, the ranking order was: *P. granatum* < *P. roxburghii* < *S. chirata* < *T. cordifolia* < *T. corniculata*. In case of combination prepared by mixing these five ingredients, the minimum 50% inhibitory concentration of 55.271 μg/ml was recorded with the highest percentage reduction i.e. 95.84% at 1000 μg/ml concentration. Additional file 1: Table S2.

#### Monitoring oxidative stress by measurement of lipid peroxides

The protection contributed by the plant extracts against oxidative deterioration was explored by inactivation of free radical chain reaction. The concentration dependent percentage of peroxyl radicals scavenged by each plant extract has been shown in Fig. 1c. The IC_50_ of 102.749 μg/ml featured *P. roxburghii* leaves extract to be a promising scavenger of peroxyl radicals as compared to *P. granatum*, *T. cordifolia* and *T. corniculata*. *S. chirata* whole herb extract showed the least effectiveness against peroxyl radicals with 50% inhibitory concentration of 179.124 μg/ml. According to the IC_50_ values displayed by the extracts, the extracts were arranged as: *P. roxburghii* < *P. granatum* < *T. corniculata* < *T. cordifolia* < *S. chirata*. It was elucidated that the combination presented the lowest IC_50_ of 82.271 μg/ml, indicating it as the most active scavenger of peroxyl radicals. Additional file 1: Table S3.

#### Reduction of cupric ions

The investigation of protective action of extracts was carried out by observing the reduction of cupric ions. As mentioned in Fig. 1d, IC_50_ of 150.551 μg/ml represented the appreciable efficacy of *P. grantum* to reduce the cupric ions and *T. corniculata* seed extract was reported to have the lowest percentage reduction with highest IC_50_ (802.174 μg/ml). The ability to reduce cupric ions by five plants was found to be in order as: *P. granatum* > *P. roxburghii* > *T. cordifolia* > *S. chirata* > *T. corniculata*. Whereas, the herbal combination presented the best antioxidant activity as revealed by its lowest IC_50_ of 96.356 μg/ml. Additional file 1: Table S4.

#### Chelation potential for Fe^2+^ ions

The increase in percentage chelation of ferrous ions by the five different plant extracts was observed to be proportional to the concentration and is illustrated in Fig. 1e. The lowest IC_50_ of 101.341 μg/ml by *P. granatum* among all plant extracts elucidated its potent chelating power ability against ferrous ions. *T. cordifolia* was revealed to hold the least chelation efficacy with IC_50_ of 361.265 μg/ml. The chelating power ability of five plants was found to be in order as: *P. granatum* > *S. chirata* > *T. corniculata* > *P. roxburghii* > *T. cordifolia*. Further, the herbal combination indicated the lowest IC_50_ of 93.143 μg/ml presenting it to be the most active chelator of ferrous ions in comparison to single species. Additional file 1: Table S5.

#### Cell-based antioxidant protection in erythrocytes

The propensity of plant extracts and herbal combination to inhibit the oxidative damage within the erythrocytes was analyzed and the results are presented in Fig. 1f. *P. granatum* peel extract demonstrated the lowest 50% inhibitory concentration of 95.559 μg/ml in comparison to *P. roxburghii* (167.304 μg/ml)*, S. chirata* (230.324 μg/ml)*, T. cordifolia* (293.281 μg/ml) and *T. corniculata* (392.253 μg/ml*)*. The lowest IC_50_ of *P. granatum* indicated its high capability in protecting the cells from oxidative damage and the highest IC_50_ value of *T. corniculata* pointed towards its least inhibitory potential against oxidative stress. Considering the IC_50_ values, the ranking order was: *P. granatum* < *P. roxburghii* < *S. chirata* < *T. cordifolia* < *T. corniculata*. In case of combination, the minimum 50% inhibitory concentration of 66.754 μg/ml was recorded with the highest percentage inhibition of 99.18% at 1000 μg/ml. Additional file 1: Table S6.

Collectively the results shown in Fig. 1 indicate that the combination of herbs provide increased antioxidant activity as compared to individual plants in all assays.

### Phenolic and flavonoid content

As shown in Table 3, *P. granatum* contained 72.9 mg GAE/100 mg dry weight of extract in phenolic content assay followed by *S. chirata*, *P. roxburghii*, *T. cordifolia* and *T. corniculata* in decreasing order respectively. Herbal combination showed the highest amount of phenolic content with 86.8 mg GAE/100 mg dry weight of extract. The flavonoid content was 66.4 mg CE/100 mg dry weight of extract in *P. granatum* followed by *P. roxburghii*, *S. chirata*, *T. corniculata* and *T. cordifolia* in descending order respectively. The flavonoid content was highest in the herbal combination with amount of 78.9 mg CE/100 mg dry weight of extract.

**Table 3:** Phenolic and flavonoid content in different plant extracts and herbal combination

S. No.	Extract	Phenolic Content mg GAE/100 mg dry weight of extract (Mean ± SE)	Flavonoid Content mg CE/100 mg dry weight of extract (Mean ± SE)
1.	*P. granatum*	72.9 ± 0.53	66.4 ± 0.26
2.	*P. roxburghii*	63.8 ± 0.24	55.8 ± 0.39
3.	*S. chirata*	68.5 ± 0.72	41.2 ± 0.71
4.	*T. cordifolia*	50.6 ± 0.39	33.2 ± 0.54
5.	*T. corniculata*	47.7 ± 0.68	34.7 ± 0.63
6.	Herbal combination	86.8 ± 0.48	78.9 ± 0.41

### HPLC-PDA analysis

The chromatographic method developed and used in this study has been found to be sensitive and accurate as observed by low detection and quantitative limits with high linearity (Table 4). The intraday and interday precision and accuracy of method showed good reproducibility and repeatability as shown in Table 5. The method was analytically precise in simultaneous detection and quantification of 11 polyphenols. Additional file 2: Figures S1–S6.

**Table 4:** HPLC-PDA method detection and quantification limit of eleven polyphenolic compounds with linearity

Compound name	Linearity range (μg/ml)	Detection limit (μg/ml)	Quantitative limit (μg/ml)	Regression equation, f(x)	Correlation coefficient (r)
Gallic acid	10.4–667	0.0109	0.0330	7.01 × 10^−5^(*x*)	0.9998
Catechin	10.4–667	0.0650	0.197	4.19 × 10^−4^(*x*)	0.9999
Chlorogenic acid	5.21–333	0.0202	0.0612	1.31 × 10^−4^(*x*)	0.9999
Epicatechin	10.4–667	0.0408	0.124	2.63 × 10^−4^(*x*)	0.9999
Caffeic acid	5.21–333	0.0111	0.0337	7.19 × 10^−5^(*x*)	0.9999
Umbelliferone	5.21–333	0.0330	0.100	2.14 × 10^−4^(*x*)	0.9999
*p*-Coumaric acid	5.21–333	0.00528	0.0160	3.41 × 10^−5^(*x*)	0.9999
Rutin	10.4–667	0.0398	0.121	2.58 × 10^−4^(*x*)	0.9999
Ellagic acid	5.21–333	0.0336	0.102	2.20 × 10^−4^(*x*)	0.9999
Quercetin	5.21–333	0.0213	0.0646	1.37 × 10^−4^(*x*)	0.9999
Kaempferol	5.21–333	0.0659	0.200	4.26 × 10^−4^(*x*)	0.9996

**Table 5:** Intraday and interday precision and accuracy of HPLC-PDA method with mixture of polyphenolic compounds injected at highest concentration

Precision^a^ and accuracy^b^	Polyphenolic compounds										
	GA^1^	CAT^2^	ChA^3^	ECAT^4^	CA^5^	UMB^6^	CoA^7^	RUT^8^	EA^9^	QUE^10^	KAE^11^
Intraday (*n* = 9)											
Day 1											
Average area	9,498,108	1,587,143	2,536,193	2,519,193	4,619,983	1,549,082	9,747,582	2,579,650	1,499,363	2,429,518	782,051
±SD	15,273	15,413	18,257	20,705	34,228	15,372	66,280	14,123	11,745	8846	2835
%RSD	0.161	0.971	0.720	0.822	0.741	0.992	0.680	0.547	0.783	0.364	0.363
%Recovery	99.9	99.8	99.7	99.4	99.7	99.5	99.7	99.8	99.0	99.9	99.9
Day 2											
Average area	9,488,221	1,589,559	2,529,570	2,519,830	4,625,180	1,551,030	9,751,220	2,579,215	1,496,578	2,428,618	782,443
±SD	24,151	12,751	24,681	22,101	37,601	16,210	68,152	12,489	12,930	9764	3956
%RSD	0.255	0.802	0.976	0.877	0.813	1.05	0.699	0.484	0.864	0.402	0.505
%Recovery	99.8	99.9	99.4	99.4	99.8	99.6	99.8	99.8	98.8	99.8	100
Day 3											
Average area	9,501,364	1,586,062	2,541,010	2,522,814	4,629,476	1,548,746	9,743,617	2,582,113	1,503,104	2,432,157	782,007
±SD	16,429	14,829	24,319	24,128	34,215	14,814	74,613	12,056	13,689	7955	2245
%RSD	0.173	0.935	0.957	0.956	0.739	0.957	0.766	0.467	0.911	0.327	0.287
%Recovery	99.9	99.7	99.9	99.5	99.9	99.4	99.7	99.9	99.2	100	99.9
Interday (*n* = 27)											
3 Days with 9 replicates per day											
Average area	9,495,898	1,587,588	2,535,591	2,520,612	4,624,880	1,549,619	9,747,473	2,580,326	1,499,682	2,430,098	782,167
±SD	14,065	14,331	25,336	23,613	36,348	16,041	71,681	13,849	13,280	9962	3142
%RSD	0.148	0.903	0.999	0.937	0.786	1.04	0.735	0.537	0.852	0.410	0.401
%Recovery	99.8	99.8	99.6	99.4	99.8	99.5	99.7	99.9	99.0	99.9	100

^1^Gallic acid; ^2^Catechin; ^3^Chlorogenic acid; ^4^Epicatechin; ^5^Caffeic acid; ^6^Umbelliferone; ^7^*p*-Coumaric acid; ^8^Rutin; ^9^Ellagic acid; ^10^Quercetin; ^11^Kaempferol; ^a^%RSD; ^b^%Recovery

The assessment of the eleven polyphenols viz.*,* gallic acid, catechin, chlorogenic acid, epicatechin, caffeic acid, umbelliferone, coumaric acid, rutin, ellagic acid, quercetin and kaempferol with good resolution was conducted to strengthen the results obtained in in vitro antioxidant analysis. Among five plant extracts, *P. granatum* was observed to contain maximum amount (79.75 μg/mg) of total polyphenols followed by *P. roxburghii* as shown in Table 6. The highest composition of 44.16 μg/mg of kaempferol was detected in *P. granatum* peel extract. The maximum content of kaempferol (22.78 μg/mg), ellagic acid (19.22 μg/mg), gallic acid (1.46 μg/mg), quercetin (8.34 μg/mg) was noted in *P. roxburghii*, *S. chirata*, *T. cordifolia* and *T. corniculata* respectively. Based on the chromatograms, the herbal samples were ranked in accordance to their polyphenolic amount as: *P. granatum* > *P. roxburghii* > *S. chirata* > *T. corniculata* > *T. cordifolia*.

**Table 6:** HPLC-PDA quantification of polyphenolic compounds in plant extracts

Retention time	Compound	PG^a^	PR^b^	SC^c^	TCord^d^	TCorn^e^
		Concentration (μg/mg)				
2.512	Gallic acid	5.66	11.61	19.14	1.46	1.92
3.912	Catechin	2.46	10.65	0.23	0.53	5.04
4.630	Chlorogenic acid	3.69	1.39	0.08	0.44	0.05
6.165	Epicatechin	3.62	8.74	18.21	0.09	N.D.
6.889	Caffeic acid	0.15	N.D.	N.D.	0.02	N.D.
9.761	Umbelliferone	0.66	0.44	0.95	N.D.	4.78
10.360	Coumaric acid	N.D.	N.D.	0.62	N.D.	0.25
14.915	Rutin	N.D.	N.D.	3.60	N.D.	2.30
15.617	Ellagic acid	14.36	6.37	19.22	0.51	8.15
16.486	Quercetin	4.99	1.99	N.D.	0.53	8.34
17.224	Kaempferol	44.16	22.78	N.D.	1.01	1.93
Total Polyphenols		79.75	63.97	62.05	4.59	32.76

N.D. - Not detected; ^a^*Punica granatum*; ^b^*Putranjiva roxburghii; *^c^*Swertia chirata;* ^d^*Tinospora cordifolia*; ^e^*Trigonella corniculata*

### Principal component analysis

**Fig. 2:**
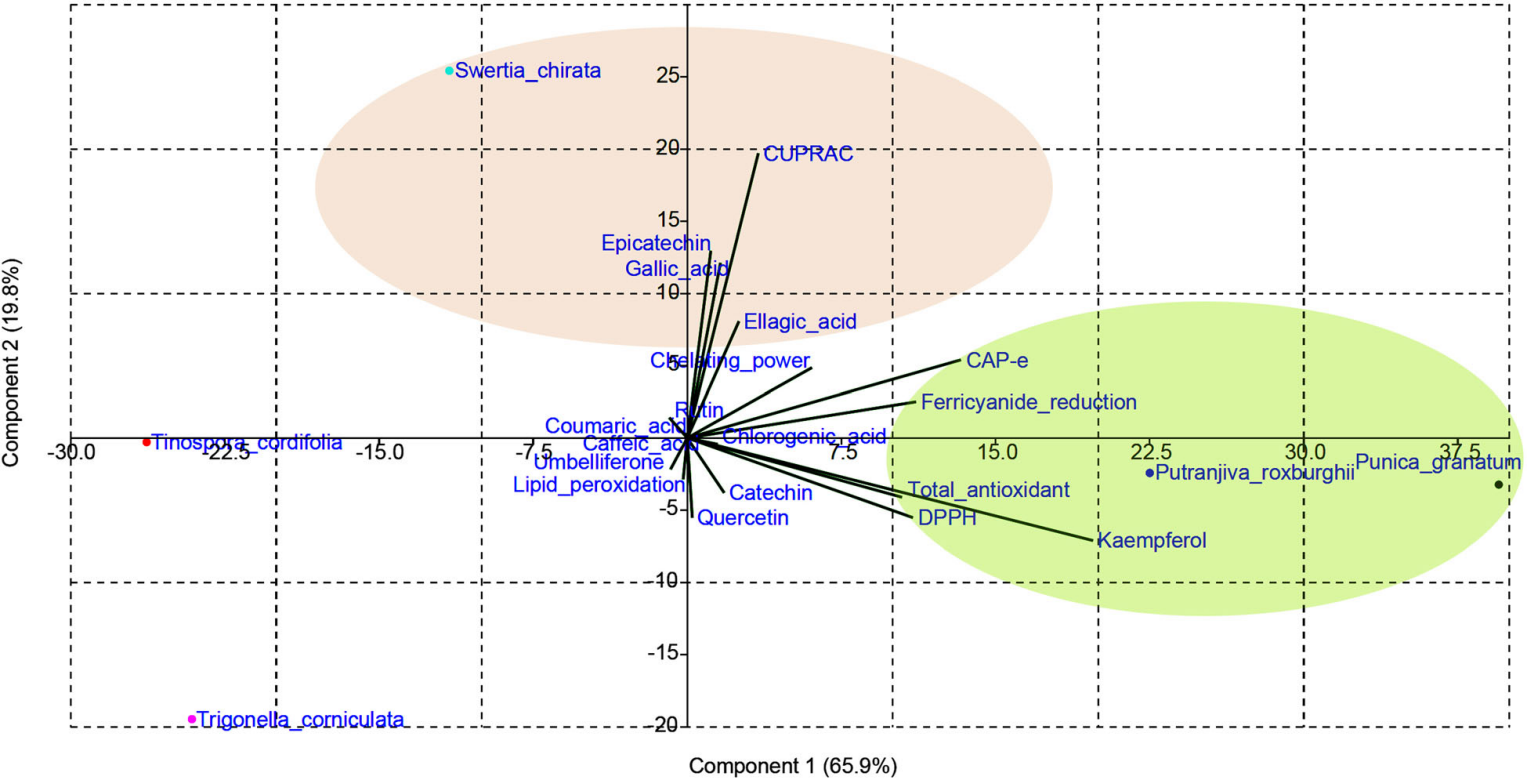
Reduction of multidimensional variables by Principal Component Analysis (PCA) for five medicinal plants

Principal component analysis (PCA) was performed on the multidimensional variables of five plants. The PCA reduced the variables into two main principal components with component 1 and 2 explaining 65.9 and 19.8% variability respectively (Fig. 2). Total of 85.7% variability was explained by both component 1 (PC1) and component 2 (PC2), with PC1 being the prominent one. PC1 was positively associated with almost all variables except rutin, umbelliferone and caffeic acid. It was found that PC1 has no association with lipid peroxidation assay. Overall, PC1 explained high variability through kaempferol, DPPH, ferricyanide ion reduction, total antioxidant and CAP-e assay. Whereas, PC2 was found to be positively associated with ferricyanide ion reduction assay, CUPRAC, chelating power assay, gallic acid, epicatechin, coumaric acid, rutin and ellagic acid. The highest variability of PC2 can be explained by CUPRAC, gallic acid, epicatechin and ellagic acid. On the basis of PC scores, the herbal combination was prepared in proportion of 33, 27, 25, 10 and 5% for *P. granatum*, *P. roxburghii, S. chirata*, *T. cordifolia* and *T. corniculata* respectively. Further, this combination was evaluated for its antiproliferative activity against different human cell lines as follows.

### Assessment of antiproliferative potential of herbal combination

#### Resazurin cell viability assay

**Fig. 3:**
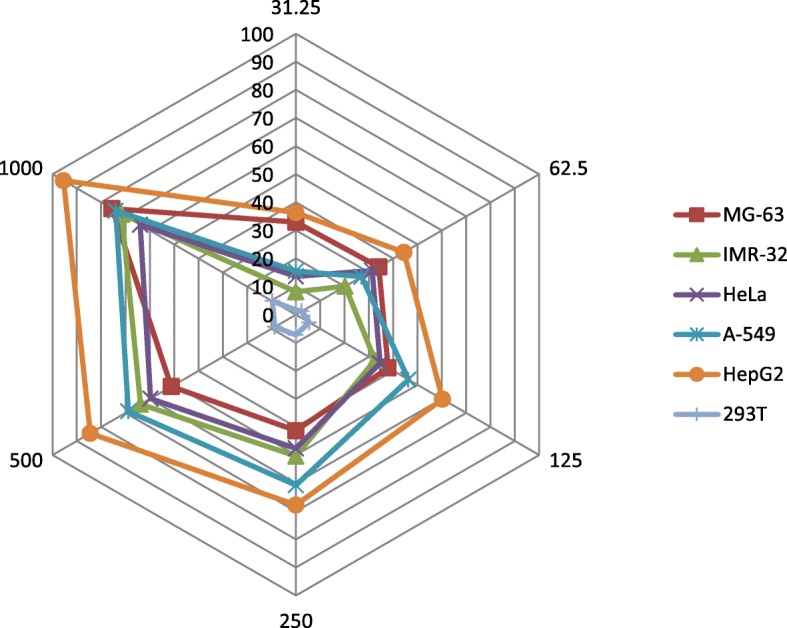
Radial graph of growth inhibitory activity of herbal combination against different human cell lines

The growth inhibitory effect of herbal combination was carried out using different malignant cells viz. MG-63, IMR-32, HeLa, HepG2 and A-549. The growth inhibitory ability of combination was also evaluated on the non-malignant 293 T (embryonic kidney) cells and results were compared with malignant cells as shown in Table 7. The herbal combination was screened against a panel of human derived cell lines of different histological origin by reduction of resazurin as a fluorometric marker for the estimation of cellular viability. The effectiveness demonstrated by combination to inhibit the proliferation of different cancer cells based on IC_50_ values was: HepG2 > A-549 > IMR-32 > HeLa > MG-63. It was observed that among all malignant cells the combination significantly inhibited the proliferation of HepG2 cells with lowest IC_50_ of 75.864 μg/ml. The inhibitory effect of the combination was significantly prominent against HepG2 cells at each tested concentration as depicted in Fig. 3. Also, the combination was seen to be non-toxic towards non-malignant embryonic kidney (293 T) cells as it showed relatively high IC_50_ value of 9.32 × 10^9^ μg/ml.

**Table 7:** Percentage growth inhibitory effect of herbal combination against different human cell lines

Conc. (μg/ml)	MC^a^					NMC^b^
	MG-63	IMR-32	HeLa	A-549	HepG2	293 T
	Inhibition (% ± SE)					
31.25	32.947 ± 0.809	08.141 ± 1.339	13.583 ± 0.676	15.487 ± 0.366	36.338 ± 0.972	1.695 ± 0.676
62.5	34.090 ± 0.024	20.261 ± 0.235	31.336 ± 0.821	27.051 ± 1.012	44.376 ± 0.570	2.452 ± 0.567
125	37.926 ± 0.280	32.510 ± 0.284	34.619 ± 0.911	46.275 ± 0.411	60.264 ± 0.247	5.510 ± 0.440
250	41.271 ± 0.512	50.335 ± 0.598	47.664 ± 0.903	60.716 ± 0.171	67.763 ± 0.027	7.206 ± 0.621
500	51.194 ± 0.537	63.810 ± 0.402	59.736 ± 0.671	68.883 ± 0.230	84.514 ± 0.021	8.689 ± 0.813
1000	75.440 ± 0.368	71.598 ± 0.257	63.992 ± 0.677	74.007 ± 0.106	95.448 ± 0.011	9.658 ± 0.424
Regression equation	y = 0.043x + 31.28	y = 19.19ln(x) - 58.24	y = 14.43ln(x) - 32.89	y = 17.82ln(x) - 43.52	y = 17.45ln(x) - 25.54	y = 2.482ln(x) - 6.976
R^2^	0.995	0.991	0.970	0.964	0.991	0.971
IC_50_ (μg/ml)	435.349	281.586	312.399	189.025	75.864	9.322 × 10^9^
Camptothecin (10 μM)	81.86	65.68	71.60	68.07	68.13	22.62

^a^Malignant cells; ^b^Non-malignant cell

#### Microscopic analysis of cellular and nuclear morphology

**Fig. 4:**
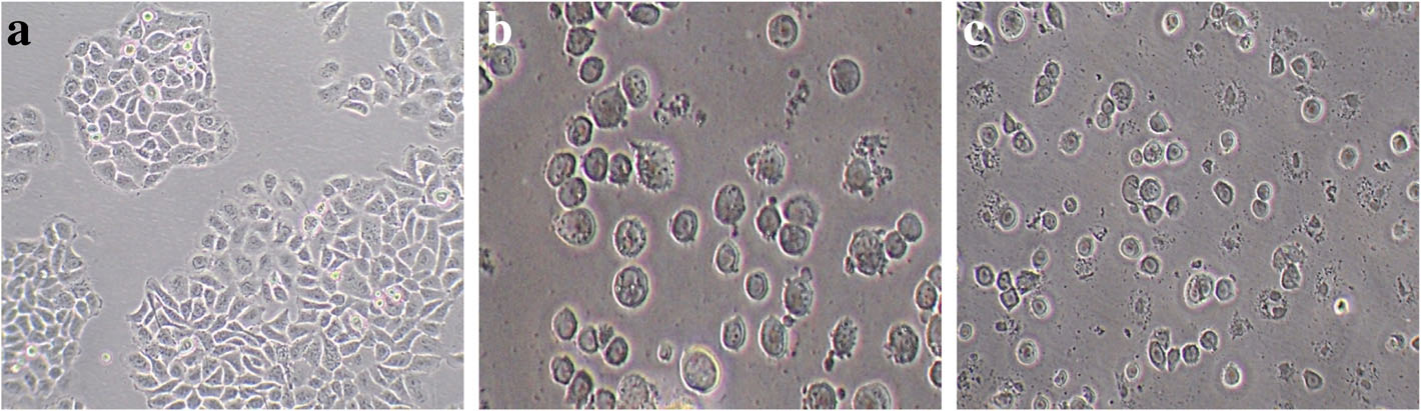
Phase contrast microscopy (**a**) HepG2 cells with no treatment (**b**) HepG2 cells treated with 10 μM camptothecin (**c**) HepG2 cells treated with IC_50_ (75.864 μg/ml) of the herbal combination

**Fig. 5:**
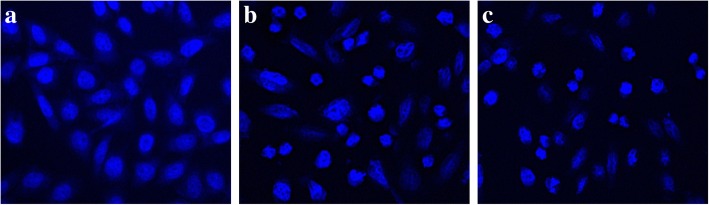
Confocal microscopy (**a**) HepG2 cells with no treatment (**b**) HepG2 cells treated with 10 μM camptothecin (**c**) HepG2 cells treated with IC_50_ (75.864 μg/ml) of the herbal combination

The treated and untreated HepG2 cells were observed under phase contrast microscope to visualize morphological changes as shown in Fig. 4a-c. It was observed that the morphological characteristics of herbal combination treated HepG2 cells were considerably changed. Under phase contrast microscope, the treated cells demonstrated destruction of HepG2 monolayer with reduction in cell population. It also resulted in cell shrinkage, cell rounding and formation of apoptotic bodies. Subsequently, the changes in nuclear morphology were also observed under confocal microscope as shown in Fig. 5a-c. The confocal observations revealed substantial changes in HepG2 nuclei like fragmentation, shrinkage and formation of apoptotic bodies.

#### Reactive oxygen species (ROS) generation

**Fig. 6:**
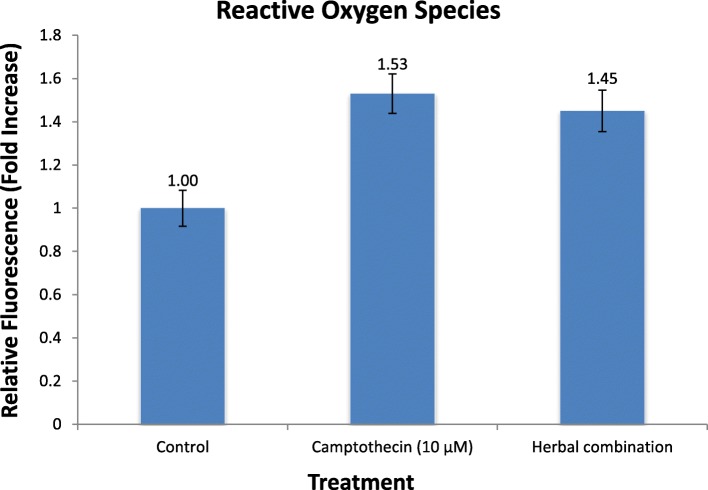
Reactive oxygen species generation in HepG2 cells after treatment with IC_50_ value of herbal combination

The reactive oxygen species (ROS) stress has been observed to be higher in cancer cells as compared to normal cells. The low to moderate ROS levels may facilitate cancer cell proliferation, whereas higher ROS generation may induce cell death. The ROS generation facilitates depolarization of mitochondria and may induce apoptosis. The fluorescence value of 2′-7′dichlorofluorescein diacetate (DCF-DA) in the untreated HepG2 cells was considered as one and the relative fluorescence increase in treatment groups was then determined. The IC_50_ treated HepG2 cells showed increase in the relative fluorescence by 1.45 fold, comparable to increase in the camptothecin (1.53 fold) treated cells, with respect to the untreated cells (Fig. 6). The increased generation of ROS in the treated HepG2 cells suggest apoptosis of hepatocellular carcinoma.

#### Mitochondrial membrane potential (MMP)

The decrease in mitochondrial membrane potential (MMP) is a critical modulatory step in intrinsic pathway of cells undergoing apoptosis. The rhodamine intensity of control cells was selected as 100% indicating the significant activity of the untreated HepG2 cells and relative intensity in other treatment groups was then calculated. The IC_50_ treated HepG2 cells showed 12.5% decrease in rhodamine intensity, comparable to the camptothecin treated cells with 16.7% decrease in rhodamine intensity, with respect to the untreated cells. The experiment revealed probable apoptotic cell death of HepG2 cells owing to decrease in MMP leading to loss in mitochondrial integrity due to shift in transmembrane potential in hepatoma cells. Additional file 1: Table S7, Additional file 2: Figure S7.

## Discussion

In recent years, considerable effort is diverted towards understanding the functional significance of the plant derived extracts and efficacious chemicals in clinical practice. The most active principles are present in the plant-based diet of humans and it has been viewed as a potentially suitable method for effective combating process against diseases, however, actual usefulness of this process is questionable due to very low amount of active agents in natural diet [23]. Thus, natural combination of active principles in the form of herbal formulations may provide better means to deal with these problems. In the present communication, we employed water as a solvent for extraction purpose instead of other organic solvents which raise concerns for their applicability in humans. Moreover, very few studies reporting herbal efficacy in the literature have been conducted using aqueous extracts. In the present study, the comparative antioxidant efficacy and the polyphenolic composition of *Punica granatum* L., *Putranjiva roxburghii* Wall., *Swertia chirata* Buch.-Ham., *Tinospora cordifolia* (Willd.) Miers and *Trigonella corniculata* L. were assessed. The complete data set for antioxidant activity and HPLC-PDA quantification data of phenolic compounds were analyzed statistically with principal component analysis (PCA) for preparation of multi-herbal combination.

The PCA divided the five medicinal plants into three groups, in which the first group consisting of *P. granatum* peel and *P. roxburghii* leaves explained the highest antioxidant activity strongly correlated with the presence of high amount of kaempferol. Structurally, the efficient radical scavenging ability of kaempferol appeared to be due to the enhanced planarity caused by the presence of 3-OH group on chroman ring [24]. The first PCA group containing the fruit peel extract of *P. granatum* was found to be the most superior in ferricyanide ion reduction assay with IC_50_ value of 63.43 μg/ml closely followed by DPPH assay with 64.20 μg/ml IC_50_ value and CAP-e assay showed 95.56 μg/ml IC_50_ value among all antioxidant assays. However, at 1000 μg/ml concentration *P. granatum* displayed the highest antioxidant activity of 94.88% in CAP-e assay. The polyphenolic rich peel extract exerted strong antioxidative action, which might be attributed to its ability to donate hydrogen atom, enabling termination of free radical generation. Thus, indicating superior capability of this plant in other in vitro assays as well. The high reducing ability of *P. granatum* is analogous with the previous antioxidant studies on its fruit peel [25, 26]. In addition to antioxidant ability, the eco-friendly biological waste and nutraceutic resource i.e.*,* peel of *P. granatum* has been depicted to possess numerous other therapeutic actions viz.*,* anti-bacterial, anti-mutagenic, anti-neoplastic, anti-viral, anti-inflammatory, anti-hyperglycemic and hepatoprotective [27–31]. Furthermore, the second plant of the same PCA group i.e. the leaf extract of *P. roxburghii* also showed superiority in antioxidant activity for DPPH assay with 71.38 μg/ml IC_50_ value, among all antioxidant assays. With kaempferol being major active principle in it, the ability of leaf extract to scavenge DPPH radicals might be attributed to potentially strong hydrogen or electron donating capacity thus, preventing formation of reactive oxygen species. Along with the antioxidant activity, the leaf extract has been reported to possess strong analgesic, antipyretic and anti-inflammatory actions [32, 33]. Thus, PCA identified *P. granatum* peel and *P. roxburghii* leaves as the leading or primary herbs for the preparation of multi-herbal combination.

The second PCA group consisted of only one plant, *S. chirata* Buch.-Ham. The antioxidant activity of this herbal extract was correlated with gallic acid, epicatechin and ellagic acid. Structurally, the antioxidant ability of gallic acid has been due to the vicinal presence of three OH functional groups on benzene ring [24]. The radical scavenging ability of epicatechin might be due to vicinal substitution of hydroxyl groups on benzene ring (alike catechol moiety) or because of appearance of gallate esterified at OH group at position 3 in dihydropyran heterocycle ring [24]. The ellagic acid has two orthodiphenolic functionalities, which maintain the total spin density delocalization, leading to more stability of 5-O^•^ radical of compound enabling its high activity in antioxidant assays [34]. The antioxidant activity of *S. chirata* whole plant extract has been found to be more prevalent in cupric ion reduction assay with 89.8% reduction potential at 1000 μg/ml concentration. However, the lowest IC_50_ value of 137.43 μg/ml was observed in chelating power assay closely followed by ferricyanide ion reduction assay with IC_50_ value of 143.42 μg/ml. Thus, suggesting strong chelating power and hydrogen donation ability indicating high antioxidant potential in other assays as well. Therefore, at the highest concentration of 1000 μg/ml, the extract has showed good antioxidant ability in cupric ion reduction assay. The traditional utilization of *S. chirata* displays wide array of therapeutic uses against ailments like anemia, fever, gastritis, bronchial asthma, malaria, hepatitis, constipation, liver ailments, dyspepsia, epilepsy, hypertension, scanty urine, melancholia, skin diseases, ulcers, worm’s infection, mental disorders and diabetes. It also helps in maintaining bile secretion and purifies blood [35]. *S. chirata* is one of the crude raw materials for pharmaceutical industries which is used in multifarious marketed herbal preparations and is a MAP (medicinal and aromatic plant) crop [36]. The whole plant of *S. chirata* possesses sky-scraping demand and has massive prospective in the marketplace for long tenure commercialization. *S. chirata* can be recognized as a nourisher in the multi-herbal combination due to its broad spectrum of pharmacological qualities.

The third PCA group consists of *T. corniculata* L. and *T. cordifolia* (Willd.) Miers with lowest associated variables from antioxidant activity and polyphenolic compounds, thus positioned as outliers. Among all antioxidant assays, the highest detected activity of *T. corniculata* L. seed extract has been found to be more noticeable in lipid peroxidation assay with lowest IC_50_ value of 137.01 μg/ml closely followed by chelating power assay with IC_50_ value of 157.70 μg/ml. The high chelating activity for Fe^2+^ ions may be accountable for anti-lipid peroxidation ability of seed extract. Thus, the seed extract effectively averted the formation of peroxyl radicals, preventing oxidative stress. In accordance with present study, the therapeutic virtues reported for the seeds of *Trigonella spp*. comprise carminative, expectorant, aphrodisiac, anti-cancer, anti-microbial, hepatoprotective, anti-inflammatory and anti-lithigenic activities [37–39]. The seeds of *T. corniculata* are used as condiment in Indian subcontinent and have not been discovered much. Furthermore, Semalty et al. [40] reported that the seed extract of *T. corniculata* is a better scavenger of free radicals than *T. foenum-graecum*. The second plant of the same group i.e. *T. cordifolia* has also been found to be more conspicuous in lipid peroxidation assay with lowest IC_50_ value of 163.79 μg/ml. The stem extract effectively scavenged the peroxyl radicals. The scavenging potential might be attributed to its ability of donating hydrogen atom to radicals, preventing lipid peroxidation. *T. cordifolia*, reservoir of pharmacological properties, has been depicted to exhibit immunomodulatory, anti-diabetic, antioxidant, adaptogenic, hepatoprotective, and anti-tumor potential [41]. *T. corniculata* L. and *T. cordifolia* (Willd.) Miers might have served as active stimulator herbs in the multi-herbal combination and enhanced the synergistic efficacy of the prepared combination.

In this study, all of the five herbal samples exerted effective antioxidant power as the IC_50_ values were observed to be below concentration of 1000 μg/ml in different in vitro assays. This could be credited to the strong synchrony between structure of polyphenolic compounds and their electron or hydrogen donating ability. The effective antioxidant ability of five herbal green extracts against free radical induced oxidative stress might be due to the vicinal substitution of hydroxyl groups on catechol moiety, conjugation of 2,3- double bond to a 4-oxo functionality and existence of 3- and 5-hydroxyl groups on chroman ring of polyphenols [24]. The findings of the present investigation could be further step towards the green approach for extraction and understanding the comparative antioxidant potency. Moreover, the study proposes to offer key reference and provides groundwork for choosing the most apposite herbal samples. Hence, it is reasonable to suggest that the beneficial effects could be exploited better in a judiciously selected combination of the five selected herbal samples and could replace the synthetic antioxidants. Moreover, the combination of multiple components has been recognized as potentially better approach in treatment and prevention of cancers as compared to the single agent [42]. Further, the applicability of PCA may also provide a useful information regarding determination of plant groups to be exploited in a relevant manner to prepare specifically precise multi-herbal formulations in pharmaceutical industries.

Thus, with the aim to scrutinize the combinatorial antiproliferative efficiency of these plants and to substantiate their beneficial outcome, a multi-herbal combination was prepared on the basis of grouping in PCA. The prepared combination displayed high synergism in each antioxidant assay showing excellent antioxidant efficacy as compared to single herbs. At the highest concentration of 1000 μg/ml the combination showed highest antioxidant activity of 99.18% in CAP-e assay. The prepared combination was investigated for its antiproliferative potential against a panel of five human cancer cell lines MG-63, A-549, HeLa, HepG2 and IMR-32. Based on the IC_50_, combination exhibited the most potent growth inhibitory activity against HepG2 cells in resazurin cell viability assay. In present study, combination has been observed to kill the hepatoma cells. The results indicate that this is mediated through the apopotic pathway. The combination generated reactive oxygen species (ROS) in HepG2 cells and consequently led to the dissipation of mitochondrial membrane potential (MMP). The collapse in MMP may indicate apoptotic initiation in the hepatoma cells assisting release of cytochrome *c*. It may seem contradictory that herbal combination scavenged ROS in antioxidant assays in vitro whereas, its effect in HepG2 cells revealed ROS generation. However, the in vitro antioxidative potential of polyphenols via radical scavenging activity has been scientifically conceived as non-specific method [43]. Thus, radical scavenging effect cannot be extrapolated to anticancer effects. The polyphenols may exhibit differential therapeutic activeness in normal and cancer cells. Moreover, many ROS specifically functions as secondary messengers facilitating many events via ROS-driven cellular signalling in promoting and suppressing carcinogenesis [44]. The polyphenols are pleiotropic compounds as they act additively or synergistically through multiple targets against cancer cells [45]. Thus, generation of reactive oxygen species in cancer cells can be direct outcome of intracellular signalling for apoptotic induction in HepG2 cells. To support this result, herbal combination treated HepG2 cells were analyzed for morphological changes by phase contrast and nuclear alterations by confocal microscopy. The peculiar morphological changes introduced in human HepG2 cells by combination include rounding of cells, shrinkage of cytoplasmic membrane, membrane blebbing, apoptotic bodies and loss of contact with neighboring cells. These alterations displayed a good correlation with the fluorescent nuclear dye i.e. DAPI (4′,6-diamidino-2-phenylindole) staining. As deducible from confocal images, the treatment of combination augmented permeability of human hepatoma cells to DAPI dye demonstrating the presence of nuclear apoptotic bodies and chromatin condensation signifying apoptotic death of cancer cells. Also, the combination displayed extremely high IC_50_ value towards 293 T cells indicating probable non-toxic nature towards normal cells. This could represent the selectivity of multi-herbal combination for cancer cells and specifies that it would not target normal cells. In this instance polyphenols might have induced the apoptosis in HepG2 cells via multiple mechanisms. The inhibition of proteasome, mitochondrial function disruption, downregulation of MAPK, NF-κB and AP1 activation might be cause of apoptotic induction in one approach while, the upregulation of proapoptotic Bcl-2 family members and caspases with downregulation of Bcl-2, Bcl-xL and survivin might be other mechanism [43]. Generally, antioxidants are cytoprotective but in present study the antioxidant polyphenols inhibited growth of cancer cells. However, there are studies highlighting that polyphenols may not affect the normal cells but can facilitate apoptosis in cancer cells via differential pleiotropic modulatory effects on cell cycle regulation [45, 46], which might be a probable case in the current study. The disruption of mitochondrial membrane potential (MMP) causing permeability transition which leads to release of caspases might have induced the apoptosis in HepG2 cells in present study. As far as we know, the present study offers recognition of potentially new anti-hepatocarcinoma multi-herbal formulation with the combination of *Punica granatum* L. (peel), *Putranjiva roxburghii* Wall. (leaves), *Swertia chirata* Buch.-Ham. (whole herb), *Tinospora cordifolia* (Willd.) Miers (stem) and *Trigonella corniculata* L. (seed). This might be answerable to the contribution of mutual synergistic action of polyphenolic compounds as detected by HPLC analysis and presence of other bioactive constituents. The results with HepG2 cells provided the vital information for potentially new anti-hepatocarcinoma multi-herbal formulation. However, the two-dimensional (2D) planar HepG2 cell cultures may offer inconsistent outcomes due to disparity to primary hepatocytes [47]. Based on the observations of our study, it is anticipated that the combination may serve as a prospective multi-herbal formula for deeper research in in vivo hepatoprotective studies to verify its practical use.

## Conclusion

The present study uncovered the applicability of PCA in construing the antioxidant superiority of water extracted *P. granatum* L. and *P. roxburghii* Wall. as compared to that of *S. chirata* Buch.-Ham., *T. cordifolia* (Willd.) Miers and *T. corniculata* L. *P. granatum* and *P. roxburghii* were identified as primary or leading herbs, *S. chirata* as a nourisher herb and *T. cordifolia* and *T. corniculata* as stimulator herbs in prepared multi-herbal combination. Conclusively, the PCA can be utilized for reducing the multidimensional characteristics of medicinal plants into separate groups for development of polyherbal combinations. Further, on the basis of principal component analysis, the prepared multi-herbal combination showed best antiproliferative activity against HepG2 cells. Reactive oxygen species generation with decrease in mitochondrial membrane potential and microscopic studies revealed the probable apoptotic induction in HepG2 cells. The activity might be correlated to the mutual synergistic action of the polyphenolic compounds and the presence of other bioactive constituents in combination. Thus, this combination may serve as a prospective multi-herbal formula for deeper research in in vivo hepatoprotective studies to verify its practical use in future.

## Additional files

Additional file 1: Data of antioxidant activity of medicinal plant extracts and mitochondrial membrane potential. **Table S1.** Antioxidant activity of plant extracts and herbal combination in DPPH free radical scavenging assay. **Table S2.** Antioxidant activity of plant extracts and herbal combination in ferricyanide ion reduction assay. **Table S3.** Peroxyl radicals scavenging activity of plant extracts and herbal combination in lipid peroxidation assay. **Table S4.** Antioxidant activity of plant extracts and herbal combination in cupric ion reduction assay. **Table S5.** Ferrous ions chelation ability of plant extracts and herbal combination in chelating power assay. **Table S6.** Protective effect of plant extracts and herbal combination on erythrocytes in CAP-e assay. **Table S7.** Percentage decrease in mitochondrial membrane potential (MMP) in HepG2 cells after treatment with camptothecin and herbal combination as compared to fluorescence of untreated control cells (DOCX 37 kb)

Additional file 2: HPLC-PDA chromatograms of standards and plant extracts and figure for mitochondrial membrane potential. **Figure S1.** HPLC-PDA chromatogram of polyphenolic standards. **Figure S2.** HPLC-PDA chromatogram of *Punica granatum* L. (peel) extract. **Figure S3.** HPLC-PDA chromatogram of *Putranjiva roxburghii* Wall. (leaves) extract. **Figure S4.** HPLC-PDA chromatogram of *Swertia chirata* Buch.-Ham. (whole herb) extract. **Figure S5.** HPLC-PDA chromatogram of *Tinospora cordifolia* (Willd.) Miers (stem) extract. **Figure S6.** HPLC-PDA chromatogram of *Trigonella corniculata* L. (seed) extract. **Figure S7.** Percent decrease in Rhodamine intensity in HepG2 cells after treatment with IC_50_ value of Herbal combination (DOCX 2931 kb)

## Abbreviations

2D: Two-dimensional
AAE: Ascorbic acid equivalents
ANOVA: Analysis of variance
AP1: Activator protein 1
Bcl-2: B-cell lymphoma 2
Bcl-xL: B-cell lymphoma-extra large
CAP-e: Cell-based antioxidant protection in erythrocytes
CE: Catechin equivalent
CUPRAC: Cupric ion reducing antioxidant capacity
DAPI: 4′,6-Diamidino-2-phenylindole
DCF-DA: 2′-7′Dichlorofluorescein diacetate
DMEM: Dulbecco’s modified eagle’s medium
DPPH: 2–2′-Diphenyl-1-picrylhydrazyl
EDTA: Ethylenediaminetetraacetic acid
GAE: Gallic acid equivalent
HPLC-PDA: High performance liquid chromatography-photo diode array
IC_50_: Half maximal inhibitory concentration
MAP: Medicinal and aromatic plant
MAPK: Mitogen-activated protein kinase
MMP: Mitochondrial membrane potential
NF-κB: Nuclear factor kappa-light-chain-enhancer of activated B cells
PAST: Paleontological statistics
PBS: Phosphate buffered saline
PCA: Principal component analysis
RBC: Red blood cell
ROS: Reactive oxygen species
RPMI-1640: Roswell park memorial institute medium
RSD: Relative standard deviation
S/N: signal-to-noise ratio
SE: Standard error
TBARS: Thiobarbituric acid reactive substances

## References

[1] Long F, Yang H, Xu Y, Hao H, Li P (2015). A strategy for the identification of combinatorial bioactive compounds contributing to the holistic effect of herbal medicines. Sci Rep.

[2] Manach C, Scalbert A, Morand C, Rémésy C, Jiménez L (2004). Polyphenols: food sources and bioavailability. Am J Clin Nutr.

[3] Chemat F, Vian MA, Cravotto G (2012). Green extraction of natural products: concept and principles. Int J Mol Sci.

[4] Brglez Mojzer E, Knez Hrnčič M, Škerget M, Knez Ž, Bren U (2016). Polyphenols: extraction methods, antioxidative action, bioavailability and anticarcinogenic effects. Molecules.

[5] Bansal SS, Goel M, Aqil F, Vadhanam MV, Gupta RC (2011). Advanced drug delivery systems of curcumin for cancer chemoprevention. Cancer Prev Res.

[6] Plaza M, Turner C (2015). Pressurized hot water extraction of bioactives. TrAC Trends Anal Chem.

[7] Anonymous. The Ayurvedic Pharmacopoeia of India, Part-I, vol. I. Government of India: Department of ISM&H, Ministry of Health and Family Welfare; 1990.

[8] Anantha ND. Approaches to pre-formulation R and D for phytopharmaceuticals emanating from herb based traditional ayurvedic processes. J Ayurveda Integr Med. 2013;4:4–8. 10.4103/0975-9476.109542 PMC3667432 23741154

[9] Gul MZ, Bhakshu LM, Ahmad F, Kondapi AK, Qureshi IA, Ghazi IA (2011). Evaluation of *Abelmoschus moschatus* extracts for antioxidant, free radical scavenging, antimicrobial and antiproliferative activities using *in vitro* assays. BMC Complement Altern Med.

[10] Kang SH, Jeon YD, Cha JY, Hwang SW, Lee HY, Park M, Lee BR, Shin MK, Kim SJ, Shin SM, Kim DK, Jin JS, Lee YM. Antioxidant and skin-whitening effects of aerial part of *Euphorbia supina* Raf. extract. BMC Complement Altern Med. 2018;18:256. 10.1186/s12906-018-2323-5 PMC6142622 30223806

[11] Kalyana Sundaram I, Sarangi DD, Sundararajan V, George S, Sheik Mohideen S (2018). Poly herbal formulation with anti-elastase and anti-oxidant properties for skin anti-aging. BMC Complement Altern Med.

[12] Kumar S, Pandey AK (2014). Medicinal attributes of *Solanum xanthocarpum* fruit consumed by several tribal communities as food: an *in vitro* antioxidant, anticancer and anti HIV perspective. BMC Complement Altern Med.

[13] Apak R, Güçlü K, Özyürek M, Karademir SE (2004). Novel total antioxidant capacity index for dietary polyphenols and vitamins C and E, using their cupric ion reducing capability in the presence of neocuproine: CUPRAC method. J Agric Food Chem.

[14] Navanesan S, Wahab NA, Manickam S, Sim KS (2015). Evaluation of selected biological capacities of *Baeckea frutescens*. BMC Complement Altern Med.

[15] Honzel D, Carter SG, Redman KA, Schauss AG, Endres JR, Jensen GS (2008). Comparison of chemical and cell-based antioxidant methods for evaluation of foods and natural products: generating multifaceted data by parallel testing using erythrocytes and polymorphonuclear cells. J Agric Food Chem.

[16] Gupta D, Gupta RK (2011). Bioprotective properties of dragon’s blood resin: *in vitro* evaluation of antioxidant activity and antimicrobial activity. BMC Complement Altern Med.

[17] Chanthasri W, Puangkeaw N, Kunworarath N, Jaisamut P, Limsuwan S, Maneenoon K, Choochana P, Chusri S (2018). Antioxidant capacities and total phenolic contents of 20 polyherbal remedies used as tonics by folk healers in Phatthalung and Songkhla provinces, Thailand. BMC Complement Altern Med.

[18] Riss TL, Moravec RA, Niles AL, Duellman S, Benink HA, Worzella TJ, Minor L. Cell viability assays. In: Sittampalam GS, Coussens NP, Brimacombe K, et al. editors. Assay guidance manual [internet]. Bethesda (MD): Eli Lilly & Company and the National Center for Advancing Translational Sciences; 2013 (updated: July 1, 2016). p. 355–85.

[19] Alam F, Najum us Saqib Q, Waheed A. Cytotoxic activity of extracts and crude saponins from *Zanthoxylum armatum* DC. against human breast (MCF-7, MDA-MB-468) and colorectal (Caco-2) cancer cell lines. BMC Complement Altern Med. 2017;17:368. 10.1186/s12906-017-1882-1 PMC5514500 28716103

[20] Shin YJ, Kim JH, Seo JM, Lee SM, Hyon JY, Yu YS, Wee WR (2009). Protective effect of clusterin on oxidative stress-induced cell death of human corneal endothelial cells. Mol Vis.

[21] Deng S, Yuan H, Yi J, Lu Y, Wei Q, Guo C, Wu J, Yuan L, He Z (2013). Gossypol acetic acid induces apoptosis in RAW264.7 cells via a caspase-dependent mitochondrial signaling pathway. J Vet Sci.

[22] Hammer Ø, Harper DAT, Ryan PD. PAST: Paleontological statistics software package for education and data analysis. Palaeontol Electron. 2001;4:1–9.

[23] Mehta RG, Pezzuto JM (2002). Discovery of cancer preventive agents from natural products: from plants to prevention. Curr Oncol Rep.

[24] Kim DO, Lee CY (2004). Comprehensive study on vitamin C equivalent antioxidant capacity (VCEAC) of various polyphenolics in scavenging a free radical and its structural relationship. Crit Rev Food Sci Nutr.

[25] Rummun N, Somanah J, Ramsaha S, Bahorun T, Neergheen-Bhujun VS (2013). Bioactivity of nonedible parts of *Punica granatum* L.: a potential source of functional ingredients. Int J Food Sci.

[26] Barathikannan K, Venkatadri B, Khusro A, Al-Dhabi NA, Agastian P, Arasu MV, Choi HS, Kim YO (2016). Chemical analysis of *Punica granatum* fruit peel and its *in vitro* and *in vivo* biological properties. BMC Complement Altern Med.

[27] Middha SK, Usha T, Pande V (2013). A review on antihyperglycemic and antihepatoprotective activity of eco-friendly *Punica granatum* peel waste. Evid- Based Complementary Altern Med.

[28] Zahin M, Ahmad I, Gupta RC, Aqil F (2014). Punicalagin and ellagic acid demonstrate antimutagenic activity and inhibition of benzo[a]pyrene induced DNA adducts. Biomed Res Int.

[29] Modaeinama S, Abasi M, Abbasi MM, Jahanban-Esfahlan R (2015). Anti tumoral properties of *Punica granatum* (pomegranate) peel extract on different human cancer cells. Asian Pac J Cancer Prev.

[30] Gullon B, Pintado ME, Pérez-Álvarez JA, Viuda-Martos M (2016). Assessment of polyphenolic profile and antibacterial activity of pomegranate peel (*Punica granatum*) flour obtained from co-product of juice extraction. Food Control.

[31] Shaygannia E, Bahmani M, Zamanzad B, Rafieian-Kopaei M (2016). A review study on *Punica granatum* L. J Evid Based Complementary Altern Med.

[32] Reanmongkol W, Noppapan T, Subhadhirasakul S. Antinociceptive, antipyretic, and anti-inflammatory activities of *Putranjiva roxburghii* Wall. leaf extract in experimental animals. J Nat Med. 2009;63:290–6. 10.1007/s11418-009-0336-6 19387768

[33] Kumar A, Kaur R, Thind TS, Arora R, Kaur P, Arora S (2015). Relative evaluation of two euphorbiaceae plants for their hydrogen donating and hydroxyl radical scavenging activities. J Chem Pharm Res.

[34] Chen Y, Xiao H, Zheng J, Liang G (2015). Structure-thermodynamics-antioxidant activity relationships of selected natural phenolic acids and derivatives: an experimental and theoretical evaluation. PLoS One.

[35] Kumar V, Van Staden J. A review of *Swertia chirayita* (Gentianaceae) as a traditional medicinal plant. Front Pharmacol. 2016;6:308. 10.3389/fphar.2015.00308 PMC4709473 26793105

[36] Lubbe A, Verpoorte R (2011). Cultivation of medicinal and aromatic plants for specialty industrial materials. Ind Crop Prod.

[37] Skaltsa H, Petropoulos GA (2002). Chemical constituents. Fenugreek: the genus *Trigonella*.

[38] Mehrafarin A, Rezazadeh SH, Naghdi Badi H, Noormohammadi GH, Zand E. Qaderi A. A review on biology, cultivation and biotechnology of fenugreek (*Trigonella foenum-graecum* L.) as a valuable medicinal plant and multipurpose. J Med Plants. 2011;1:6–24.

[39] Yadav UC, Baquer NZ (2014). Pharmacological effects of *Trigonella foenum-graecum* L. in health and disease. Pharm Biol.

[40] Semalty M, Semalty A, Joshi GP, Rawat MSM (2009). Comparison of *in vitro* antioxidant activity of *Trigonella foenum-graecum* and *T. corniculata* seeds. Res J Phytochem.

[41] Kaur P, Robin, Makanjuola VO, Arora R, Singh B, Arora S. Immunopotentiating significance of conventionally used plant adaptogens as modulators in biochemical and molecular signalling pathways in cell mediated processes. Biomed Pharmacother. 2017;95:1815–29. 10.1016/j.biopha.2017.09.081 28968926

[42] Mehta RG, Murillo G, Naithani R, Peng X (2010). Cancer chemoprevention by natural products: how far have we come?. Pharm Res.

[43] Lewandowska H, Kalinowska M, Lewandowski W, Stępkowski TM, Brzóska K (2016). The role of natural polyphenols in cell signaling and cytoprotection against cancer development. J Nutr Biochem.

[44] Chio IIC, Tuveson DA (2017). ROS in cancer: the burning question. Trends Mol Med.

[45] Russo GL, Tedesco I, Spagnuolo C, Russo M (2017). Antioxidant polyphenols in cancer treatment: friend, foe or foil?. Semin Cancer Biol.

[46] Park HK, Han DW, Park YH, Park JC (2005). Differential biological responses of green tea polyphenol in normal cells vs. cancer cells. Curr Appl Phys.

[47] Kaur P, Robin, Mehta RG, Arora S, Singh B. Progression of conventional hepatic cell culture models to bioengineered HepG2 cells for evaluation of herbal bioactivities. Biotechnol Lett. 2018;40:881–93. 10.1007/s10529-018-2547-y 29616383

