# Supplementary material for: Development of aqueous-based multi-herbal combination using principal component analysis and its functional significance in HepG2 cells
Source: BMC Complement Altern Med. 2019 Jan 15;19:18. doi: 10.1186/s12906-019-2432-9 (PMC6334454; doi:10.1186/s12906-019-2432-9)
Supplement: Supplementary file 1 — Data of antioxidant activity of medicinal plant extracts and mitochondrial membrane potential. Table S1. Antioxidant activity of plant extracts and herbal combination in DPPH free radical scavenging assay. Table S2. Antioxidant activity of plant extracts and herbal combination in ferricyanide ion reduction assay. Table S3. Peroxyl radicals scavenging activity of plant extracts and herbal combination in lipid peroxidation assay. Table S4. Antioxidant activity of plant extracts and herbal combination in cupric ion reduction assay. Table S5. Ferrous ions chelation ability of plant extracts and herbal combination in chelating power assay. Table S6. Protective effect of plant extracts and herbal combination on erythrocytes in CAP-e assay. Table S7. Percentage decrease in mitochondrial membrane potential (MMP) in HepG2 cells after treatment with camptothecin and herbal combination as compared to fluorescence of untreated control cells (DOCX 37 kb) [file 12906_2019_2432_MOESM1_ESM.docx]

**Table S1.** Antioxidant activity of plant extracts and herbal combination in DPPH free radical scavenging assay

| **Conc. (µg/ml)** | **Gallic acid** | ***P. granatum*** | ***P. roxburghii*** | ***S. chirata*** | ***T. cordifolia*** | ***T. corniculata*** | **Herbal combination** |
| --- | --- | --- | --- | --- | --- | --- | --- |
|  | **Percentage of DPPH radicals scavenged ± SE** | | | | | | |
| 15.625 | 45.63±0.737 | 29.30±1.833 | 29.19±1.259 | 10.70±0.390 | 07.13±1.067 | 14.37±0.476 | 14.42±3.511 |
| 31.25 | 60.17±0.582 | 38.55±1.716 | 35.66±1.761 | 22.17±0.365 | 10.75±1.044 | 17.72±0.486 | 40.44±1.096 |
| 62.5 | 64.18±0.531 | 47.63±0.685 | 47.08±0.201 | 32.15±0.289 | 19.72±0.613 | 21.00±0.531 | 56.86±1.291 |
| 125 | 72.65±0.456 | 59.61±1.496 | 57.27±0.402 | 46.18±0.193 | 27.58±0.821 | 25.29±0.696 | 71.25±0.212 |
| 250 | 82.17±0.435 | 73.93±0.482 | 70.75±0.579 | 63.18±0.279 | 40.56±0.223 | 48.97±1.496 | 88.55±1.223 |
| 500 | 91.42±0.201 | 83.79±0.386 | 80.50±0.628 | 68.69±0.590 | 44.18±0.675 | 64.07±0.255 | 96.15±0.147 |
| 1000 | 98.22±0.295 | 87.08±0.056 | 88.47±0.096 | 72.20±0.111 | 57.60±0.724 | 77.33±0.243 | 98.88±0.155 |
| **Reg. Eq.** | y=12.3ln(x)+14.2 | y=14.9ln(x)-12.2 | y=15.0ln(x)-14.0 | y=15.9ln(x)-31.7 | y=12.3ln(x)-29.8 | y=3.87x^0.436^ | y= 20.4ln(x)-32.0 |
| **R^2^** | 0.989 | 0.985 | 0.994 | 0.972 | 0.982 | 0.960 | 0.948 |
| **IC_50_** | 18.483 | 64.199 | 71.379 | 171.312 | 651.780 | 354.011 | 55.433 |
| **F ratio** | 1416.279* | 378.428* | 631.364* | 4831.380* | 562.934* | 1223.329* | 411.521* |
| **HSD** | 2.372 | 5.596 | 4.330 | 1.677 | 3.789 | 3.459 | 7.479 |

**p*≤0.05

**Table S2.** Antioxidant activity of plant extracts and herbal combination in ferricyanide ion reduction assay

| **Conc. (µg/ml)** | **Gallic acid** | ***P. granatum*** | ***P. roxburghii*** | ***S. chirata*** | ***T. cordifolia*** | ***T. corniculata*** | **Herbal Combination** |
| --- | --- | --- | --- | --- | --- | --- | --- |
|  | **Percentage reduction of ferricyanide ions ± SE** | | | | | | |
| 15.625 | 25.55±0.054 | 26.57±0.255 | 18.06±0.094 | 22.43±0.261 | 10.53±0.127 | 02.98±0.213 | 27.55±1.756 |
| 31.25 | 42.02±0.020 | 46.25±0.204 | 23.45±0.369 | 28.67±0.041 | 15.51±0.305 | 07.41±0.197 | 44.78±0.406 |
| 62.5 | 60.27±0.147 | 49.49±0.143 | 35.06±0.248 | 33.39±0.054 | 32.57±0.141 | 08.35±0.108 | 52.00±0.229 |
| 125 | 66.84±0.247 | 55.84±0.394 | 59.98±0.541 | 49.22±0.094 | 46.12±0.340 | 12.71±0.159 | 64.20±0.898 |
| 250 | 71.41±0.178 | 69.43±0.154 | 69.37±0.232 | 58.51±0.089 | 57.06±0.035 | 20.98±0.328 | 70.74±0.235 |
| 500 | 86.08±0.071 | 80.92±0.020 | 76.86±0.074 | 68.25±0.114 | 63.90±0.134 | 41.74±0.876 | 83.49±0.142 |
| 1000 | 100.0±0.629 | 90.47±0.317 | 91.04±0.289 | 76.55±0.321 | 70.65±0.451 | 68.25±0.779 | 95.84±1.851 |
| **Reg. Eq.** | y=16.6ln(x)-15.7 | y=14.5ln(x)-10.1 | y=18.6ln(x)-36.2 | y=13.7ln(x)-18.2 | y=15.5ln(x)-32.7 | y=0.07x+4.6 | y=15.5ln(x)-12.2 |
| **R^2^** | 0.973 | 0.975 | 0.975 | 0.985 | 0.977 | 0.991 | 0.990 |
| **IC_50_** | 51.970 | 63.432 | 104.093 | 143.419 | 204.742 | 699.231 | 55.271 |
| **F ratio** | 9335.816* | 8292.068* | 8624.249* | 14676.300* | 8299.8839* | 2449.888* | 500.850* |
| **HSD** | 1.261 | 1.164 | 1.463 | 0.826 | 1.248 | 2.311 | 5.037 |

**p*≤0.05

**Table S3.** Peroxyl radicals scavenging activity of plant extracts and herbal combination in lipid peroxidation assay

| **Conc. (µg/ml)** | **Gallic acid** | ***P. granatum*** | ***P. roxburghii*** | ***S. chirata*** | ***T. cordifolia*** | ***T. corniculata*** | **Herbal combination** |
| --- | --- | --- | --- | --- | --- | --- | --- |
|  | **Percentage of peroxyl radicals scavenged ± SE** | | | | | | |
| 15.625 | 23.40±0.768 | 15.65±0.741 | 27.82±0.170 | 05.71±0.024 | 17.23±0.148 | 07.90±0.422 | 20.36±0.064 |
| 31.25 | 43.42±0.633 | 39.16±0.274 | 34.69±0.971 | 24.54±0.321 | 28.57±0.344 | 31.49±0.168 | 37.76±0.183 |
| 62.5 | 60.88±0.296 | 45.68±0.560 | 42.57±0.234 | 33.75±0.193 | 35.47±0.400 | 43.76±0.194 | 48.52±0.087 |
| 125 | 66.74±0.790 | 52.77±0.517 | 52.62±0.689 | 47.13±1.231 | 46.98±0.404 | 49.98±0.049 | 55.64±0.105 |
| 250 | 82.68±0.319 | 57.53±0.280 | 64.04±0.129 | 55.44±0.340 | 58.48±0.257 | 57.36±0.252 | 69.87±0.487 |
| 500 | 90.99±0.216 | 66.33±0.744 | 68.93±0.652 | 67.57±0.126 | 65.72±0.863 | 69.61±0.146 | 77.19±0.151 |
| 1000 | 94.15±0.135 | 74.83±0.121 | 76.24±0.042 | 75.10±0.088 | 72.09±0.111 | 79.93±0.049 | 85.11±0.159 |
| **Reg. Eq.** | y=17ln(x)-15.9 | y=12.6ln(x)-10.4 | y=12.1ln(x)-6.1 | y=16.3ln(x)-34.4 | y=13.5ln(x)-18.8 | y=15.8ln(x)-27.5 | y=15.2ln(x)-16.9 |
| **R^2^** | 0.956 | 0.937 | 0.992 | 0.986 | 0.992 | 0.962 | 0.984 |
| **IC_50_** | 48.553 | 122.580 | 102.749 | 179.124 | 163.791 | 137.010 | 82.271 |
| **F ratio** | 2526.92* | 1424.614* | 1194.963* | 2345.932* | 2237.268* | 12155.5* | 10705.581* |
| **HSD** | 2.495 | 2.485 | 2.546 | 2.447 | 2.071 | 1.054 | 1.069 |

**p*≤0.05

**Table S4.** Antioxidant activity of plant extracts and herbal combination in cupric ion reduction assay

| **Conc. (µg/ml)** | **Gallic acid** | ***P. granatum*** | ***P. roxburghii*** | ***S. chirata*** | ***T. cordifolia*** | ***T. corniculata*** | **Herbal Combination** |
| --- | --- | --- | --- | --- | --- | --- | --- |
|  | **Percentage reduction of cupric ions ± SE** | | | | | | |
| 15.625 | 22.41±1.318 | 12.97±0.279 | 12.76±0.073 | 14.81±0.566 | 10.83±0.476 | 11.23±0.327 | 16.60±0.511 |
| 31.25 | 28.60±0.486 | 25.53±0.421 | 18.16±0.952 | 14.62±0.076 | 13.82±0.168 | 13.29±0.435 | 22.56±0.566 |
| 62.5 | 57.75±0.896 | 32.52±0.128 | 25.86±0.820 | 20.05±0.511 | 20.32±0.804 | 16.79±1.151 | 38.46±0.390 |
| 125 | 76.87±0.628 | 40.52±0.435 | 31.11±0.207 | 25.57±0.760 | 23.29±0.638 | 20.87±0.105 | 58.91±0.707 |
| 250 | 87.17±0.897 | 53.33±0.036 | 55.88±0.594 | 38.29±0.948 | 36.37±0.456 | 26.33±1.207 | 64.26±0.663 |
| 500 | 94.08±0.956 | 79.28±0.117 | 74.31±0.603 | 65.31±0.237 | 56.36±0.597 | 37.15±0.318 | 90.25±0.738 |
| 1000 | 100±0.706 | 83.34±0.290 | 76.16±0.470 | 89.76±0.734 | 82.08±0.872 | 59.08±0.228 | 96.97±0.770 |
| **Reg. Eq.** | y=20.3ln(x)-31.1 | y=17.5ln(x)-37.7 | y=17.1ln(x)-40.7 | y=0.08x+15.9 | y=2.67x^0.488^ | y=0.05x+13.1 | y=20.7ln(x)-44.7 |
| **R^2^** | 0.939 | 0.962 | 0.938 | 0.969 | 0.985 | 0.990 | 0.978 |
| **IC_50_** | 54.896 | 150.551 | 199.066 | 431.772 | 425.351 | 802.174 | 96.356 |
| **F ratio** | 1272.071* | 8911.362* | 1913.558* | 2186.756* | 1798.604* | 614.726* | 2447.843* |
| **HSD** | 4.237 | 1.365 | 2.923 | 2.978 | 2.962 | 3.287 | 3.062 |

**p*≤0.05

**Table S5.** Ferrous ions chelation ability of plant extracts and herbal combination in chelating power assay

| **Conc. (µg/ml)** | **EDTA** | ***P. granatum*** | ***P. roxburghii*** | ***S. chirata*** | ***T. cordifolia*** | ***T. corniculata*** | **Herbal combination** |
| --- | --- | --- | --- | --- | --- | --- | --- |
|  | **Percentage of ferrous ions chelated ± SE** | | | | | | |
| 15.625 | 28.42±1.495 | 21.21±0.932 | 26.80±1.692 | 21.42±0.655 | 10.44±0.215 | 20.67±1.732 | 11.73±1.772 |
| 31.25 | 45.96±1.586 | 36.38±0.373 | 27.77±1.027 | 33.69±0.938 | 17.98±0.672 | 37.03±0.646 | 36.81±0.569 |
| 62.5 | 54.90±1.480 | 44.03±0.841 | 34.55±1.213 | 43.27±1.19 | 30.25±0.932 | 44.03±1.027 | 51.02±0.215 |
| 125 | 68.68±0.186 | 57.70±0.672 | 37.35±1.134 | 49.84±1.495 | 37.24±1.199 | 52.85±0.813 | 58.88±1.124 |
| 250 | 73.20±0.559 | 64.48±0.493 | 56.30±1.092 | 55.22±0.841 | 49.30±1.305 | 56.30±0.570 | 64.05±0.776 |
| 500 | 83.32±0.108 | 69.86±0.431 | 62.65±1.060 | 66.20±0.538 | 53.18±0.969 | 59.53±0.957 | 77.61±0.599 |
| 1000 | 88.81±0.285 | 75.35±0.388 | 74.06±0.493 | 72.66±0.469 | 60.39±0.753 | 63.94±1.269 | 82.56±0.854 |
| **Reg. Eq.** | y=14.2ln(x)-5.3 | y=12.8ln(x)-9.4 | y=11.8x^0.266^ | y=11.9ln(x)-8.5 | y=12.3ln(x)-22.6 | y=9.6ln(x)+1.2 | y=15.8ln(x)-21.7 |
| **R^2^** | 0.971 | 0.967 | 0.961 | 0.988 | 0.986 | 0.917 | 0.939 |
| **IC_50_** | 49.406 | 101.341 | 230.684 | 137.435 | 361.265 | 157.699 | 93.143 |
| **F ratio** | 440.221* | 979.072* | 260.747* | 369.073* | 402.137* | 199.061* | 647.004* |
| **HSD** | 4.960 | 3.025 | 5.548 | 4.477 | 4.477 | 5.158 | 4.642 |

**p*≤0.05

**Table S6.** Protective effect of plant extracts and herbal combination on erythrocytes in CAP-e assay

| **Conc. (µg/ml)** | **Gallic acid** | ***P. granatum*** | ***P. roxburghii*** | ***S. chirata*** | ***T. cordifolia*** | ***T. corniculata*** | **Herbal combination** |
| --- | --- | --- | --- | --- | --- | --- | --- |
|  | **Percentage inhibition of oxidative damage ± S.E** | | | | | | |
| 15.625 | 25.98±0.405 | 12.31±0.605 | 09.65±0.956 | 07.40±1.184 | 06.67±1.492 | 03.88±0.591 | 19.37±4.160 |
| 31.25 | 47.45±1.289 | 24.68±0.109 | 17.64±0.828 | 13.08±1.009 | 13.64±1.169 | 09.39±0.568 | 34.36±4.423 |
| 62.5 | 61.45±1.178 | 42.97±0.424 | 28.04±0.258 | 19.76±0.703 | 23.36±1.059 | 21.33±1.326 | 49.44±1.388 |
| 125 | 71.36±0.232 | 54.04±0.507 | 39.03±0.949 | 37.78±1.054 | 32.96±1.971 | 34.47±3.080 | 65.14±1.358 |
| 250 | 82.91±0.661 | 74.49±0.394 | 54.38±0.804 | 51.83±1.255 | 47.41±1.313 | 44.33±1.278 | 77.88±0.763 |
| 500 | 92.64±0.752 | 85.73±0.359 | 73.81±0.850 | 66.47±1.424 | 61.70±0.682 | 53.20±2.566 | 90.63±0.465 |
| 1000 | 99.31±0.383 | 94.88±0.544 | 88.15±0.358 | 77.14±0.203 | 69.52±0.528 | 63.74±0.946 | 99.18±0.527 |
| **Reg. Eq.** | y=17.1ln(x)-13.8 | y=20.7ln(x)-44.3 | y=19.3ln(x)-48.7 | y=17.9ln(x)-47.5 | y=15.9ln(x)-40.3 | y=15.0ln(x)-39.3 | y=19.2ln(x)-32.34 |
| **R^2^** | 0.971 | 0.991 | 0.980 | 0.979 | 0.988 | 0.994 | 0.993 |
| **IC_50_** | 41.836 | 95.559 | 167.304 | 230.324 | 293.281 | 392.253 | 66.754 |
| **F ratio** | 1069.319* | 4854.261* | 1463.224* | 671.711* | 362.792* | 167.802* | 145.496* |
| **HSD** | 3.836 | 2.155 | 3.681 | 5.056 | 6.073 | 8.372 | 11.791 |

**p*≤0.05

**Table S7.** Percentage decrease in mitochondrial membrane potential (MMP) in HepG2 cells after treatment with camptothecin and herbal combination as compared to fluorescence of untreated control cells

| **No. of observations** | | **Fluorescence** | | |
| --- | --- | --- | --- | --- |
|  |  | **Control** | **Treatment** | |
|  |  |  | **Camptothecin (10 µM)** | **Herbal Combination (IC_50_)** |
| **Replicate 1** |  | 274 | 229 | 238 |
|  |  | 271 | 226 | 243 |
|  |  | 281 | 228 | 242 |
| **Replicate 2** |  | 272 | 233 | 237 |
|  |  | 275 | 227 | 240 |
|  |  | 280 | 236 | 236 |
| **Replicate 3** |  | 281 | 225 | 246 |
|  |  | 277 | 230 | 245 |
|  |  | 271 | 234 | 244 |
| Mean | | 275.78 | 229.67 | 241.22 |
| Standard Deviation | | 4.15 | 3.80 | 3.63 |
| Standard Error | | 1.38 | 1.27 | 1.21 |
| **Percentage relative to control (%)** | | **100** | **83.3** | **87.5** |
