# Supplementary material for: Development of aqueous-based multi-herbal combination using principal component analysis and its functional significance in HepG2 cells
Source: BMC Complement Altern Med. 2019 Jan 15;19:18. doi: 10.1186/s12906-019-2432-9 (PMC6334454; doi:10.1186/s12906-019-2432-9)
Supplement: Supplementary file 2 — HPLC-PDA chromatograms of standards and plant extracts and figure for mitochondrial membrane potential. Figure S1. HPLC-PDA chromatogram of polyphenolic standards. Figure S2. HPLC-PDA chromatogram of Punica granatum L. (peel) extract. Figure S3. HPLC-PDA chromatogram of Putranjiva roxburghii Wall. (leaves) extract. Figure S4. HPLC-PDA chromatogram of Swertia chirata Buch.-Ham. (whole herb) extract. Figure S5. HPLC-PDA chromatogram of Tinospora cordifolia (Willd.) Miers (stem) extract. Figure S6. HPLC-PDA chromatogram of Trigonella corniculata L. (seed) extract. Figure S7. Percent decrease in Rhodamine intensity in HepG2 cells after treatment with IC50 value of Herbal combination (DOCX 2931 kb) [file 12906_2019_2432_MOESM2_ESM.docx]

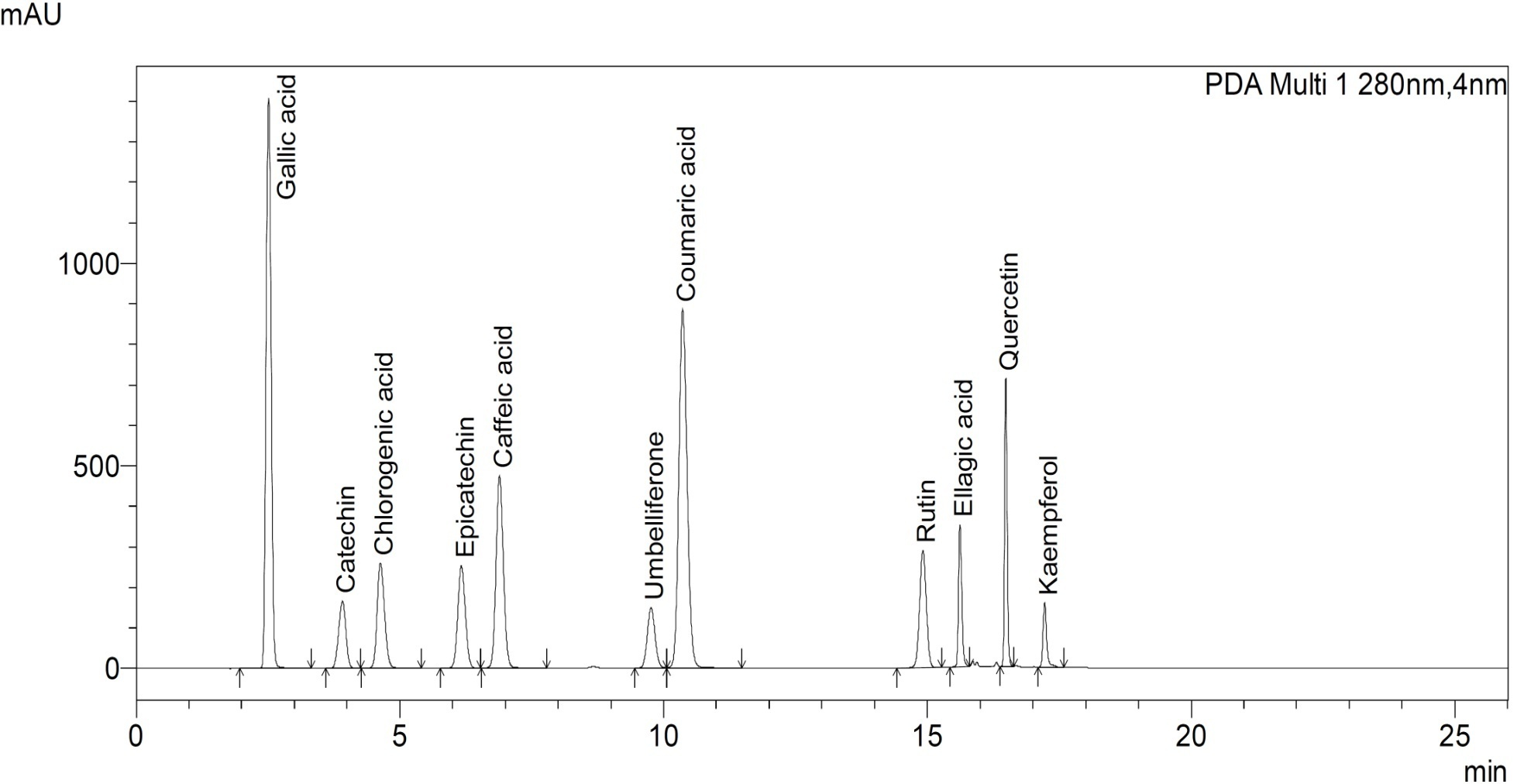


**Figure S1.** HPLC-PDA chromatogram of polyphenolic standards


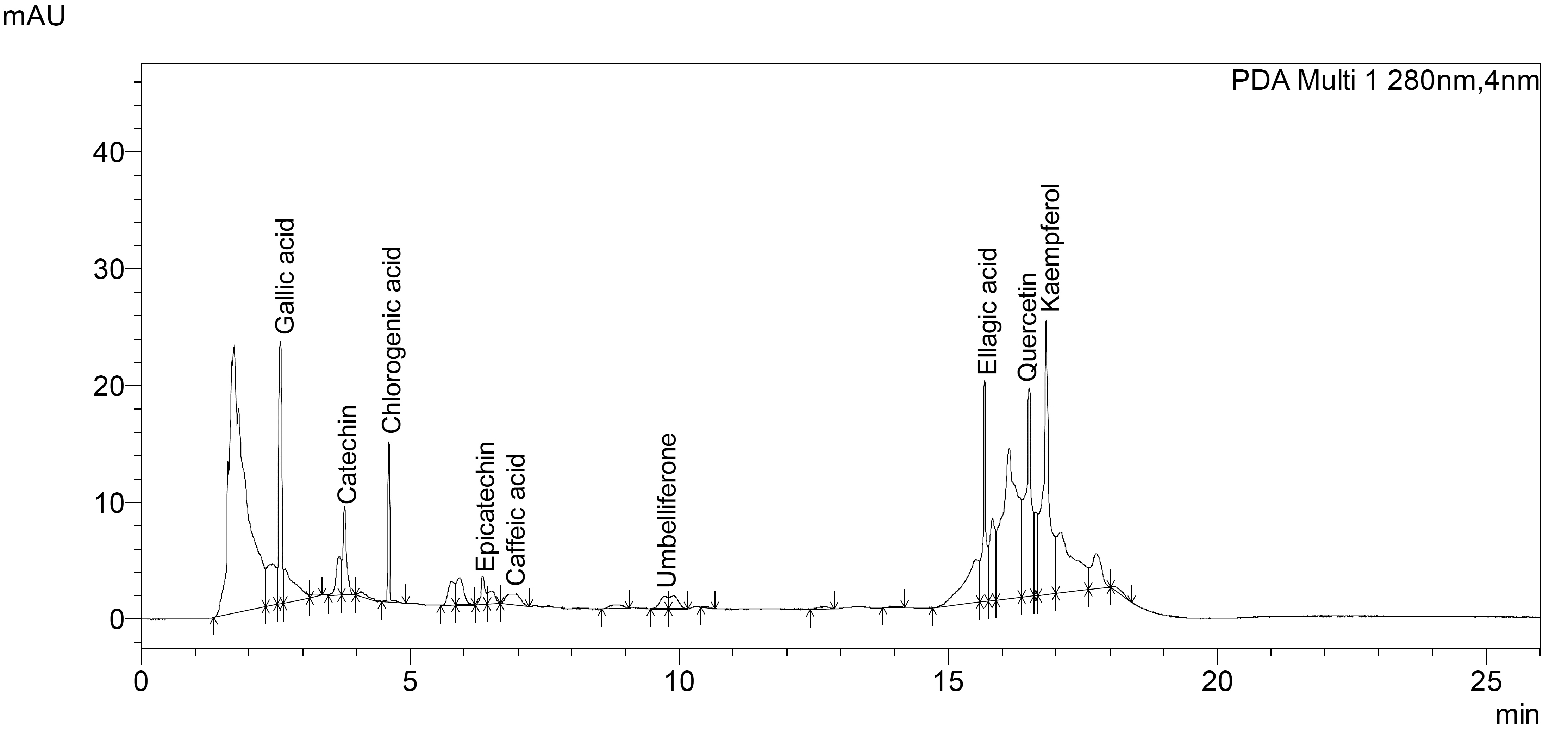


**Figure S2.** HPLC-PDA chromatogram of *Punica granatum* L. (peel) extract


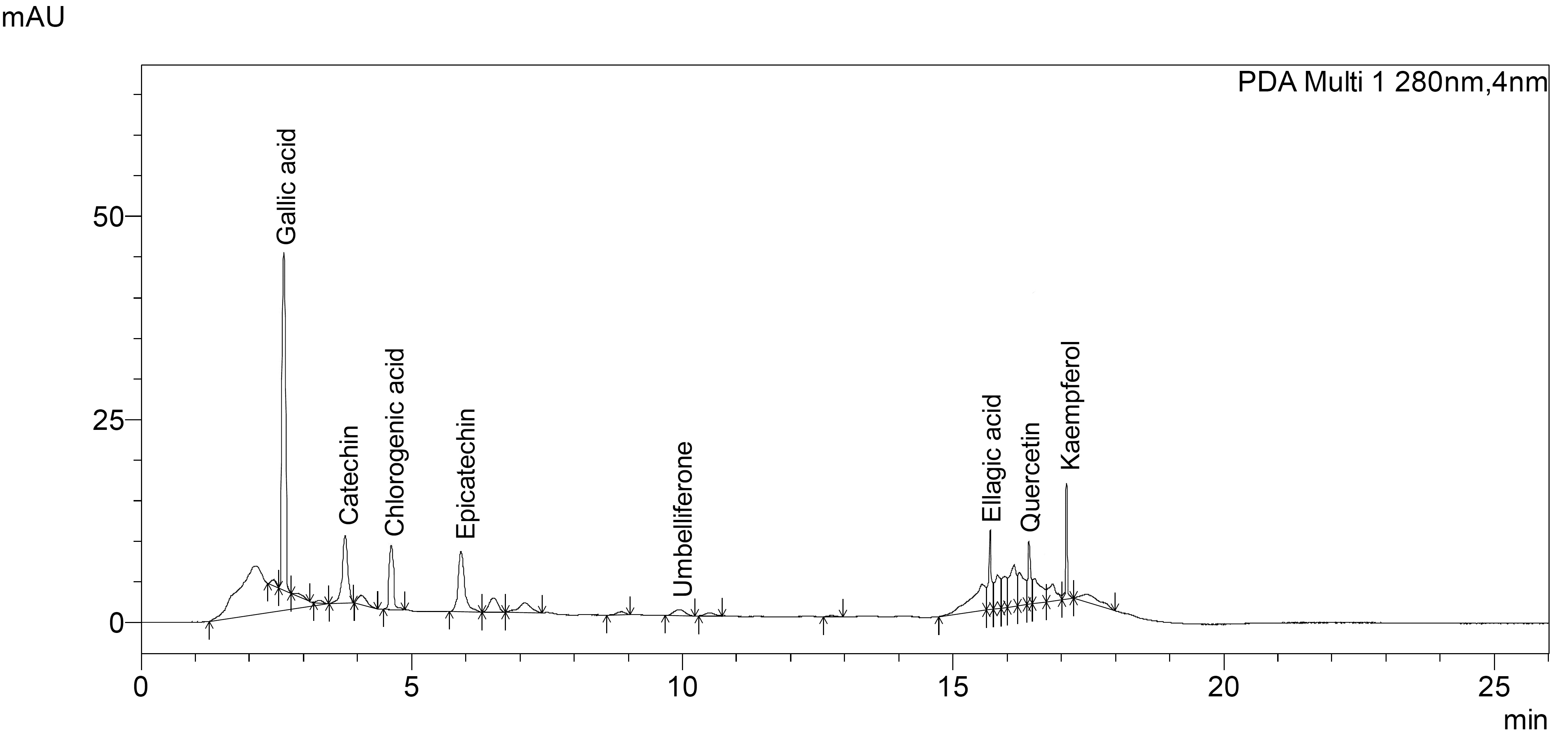


**Figure S3.** HPLC-PDA chromatogram of *Putranjiva roxburghii* Wall. (leaves) extract


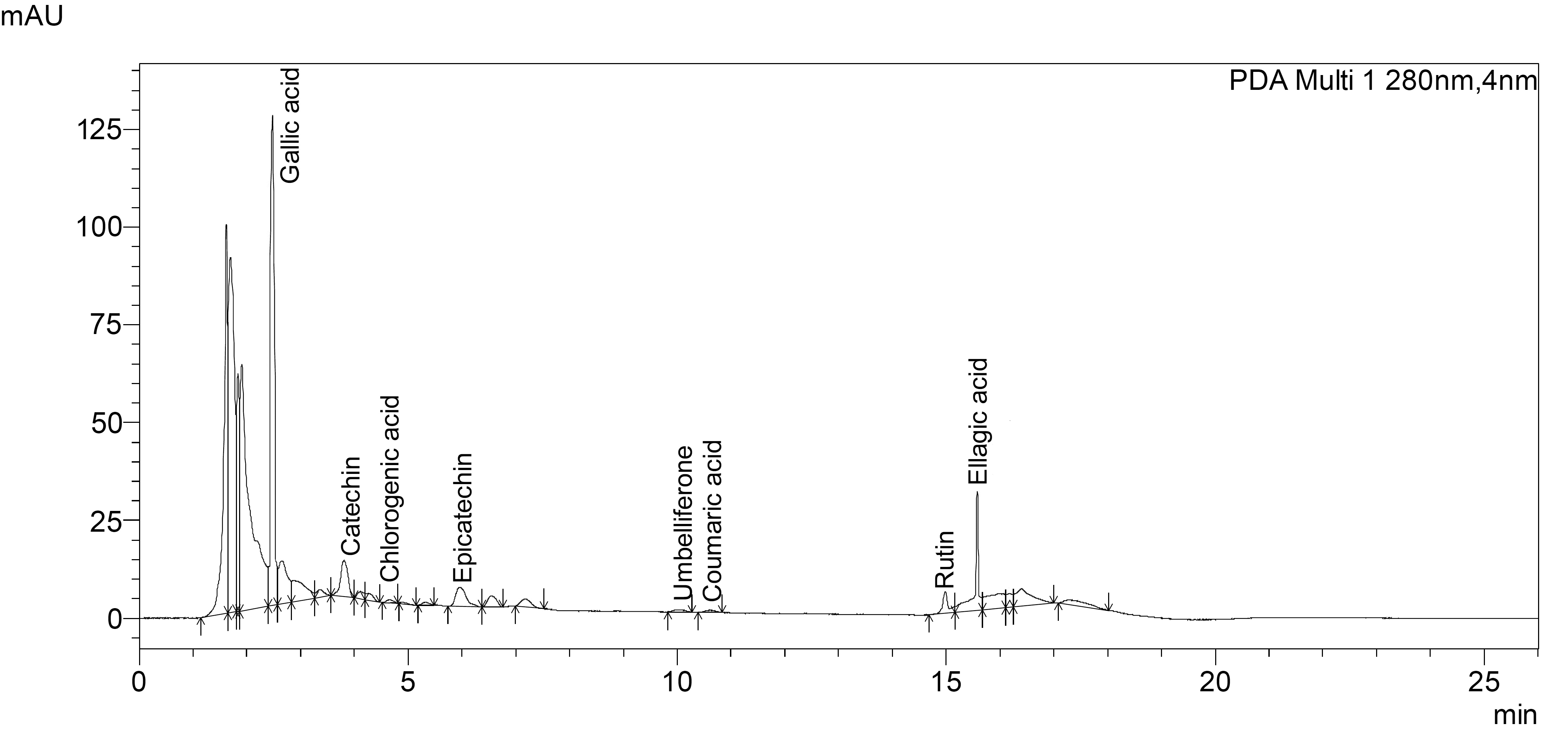


**Figure S4.** HPLC-PDA chromatogram of *Swertia chirata* Buch.-Ham. (whole herb) extract


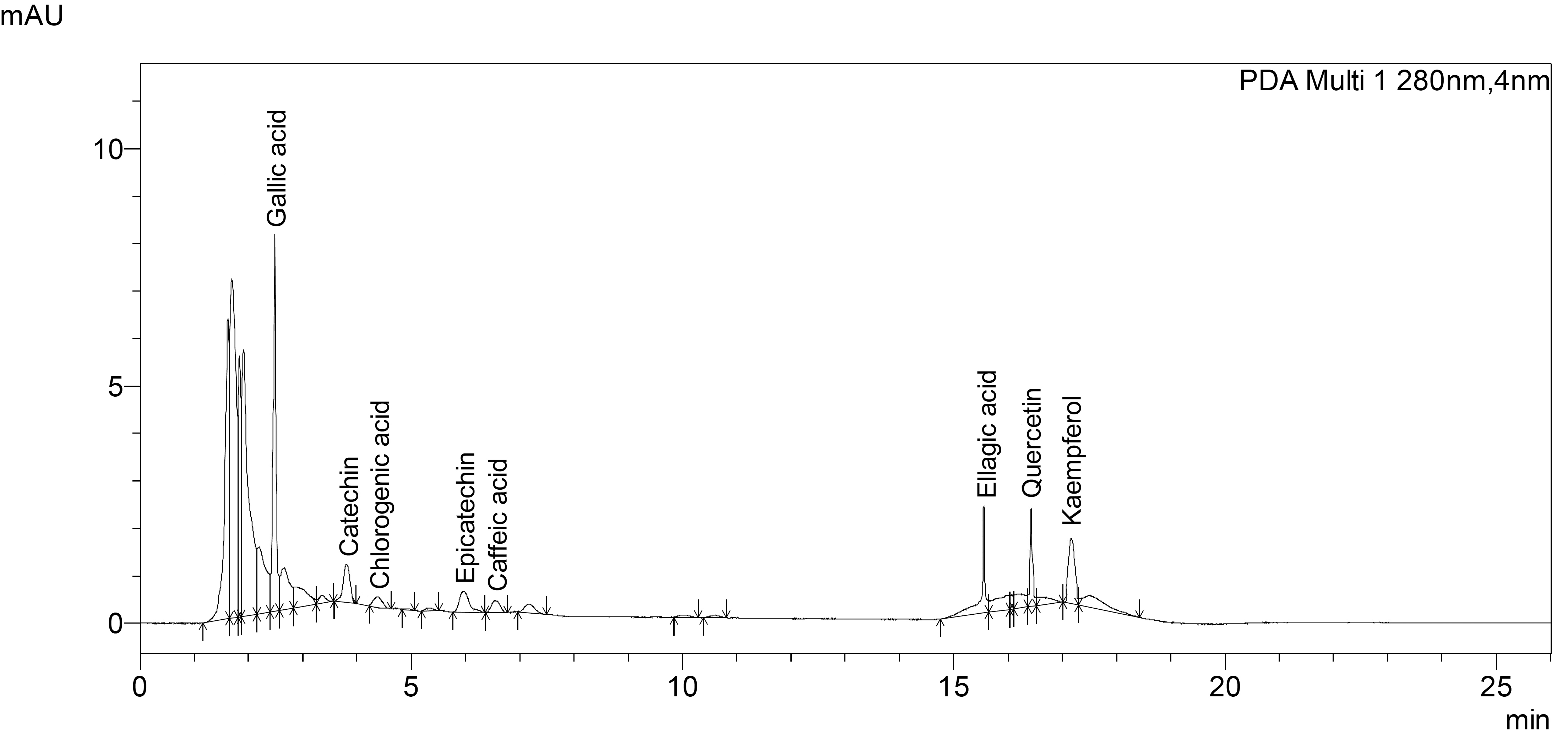


**Figure S5.** HPLC-PDA chromatogram of *Tinospora cordifolia* (Willd.) Miers (stem) extract


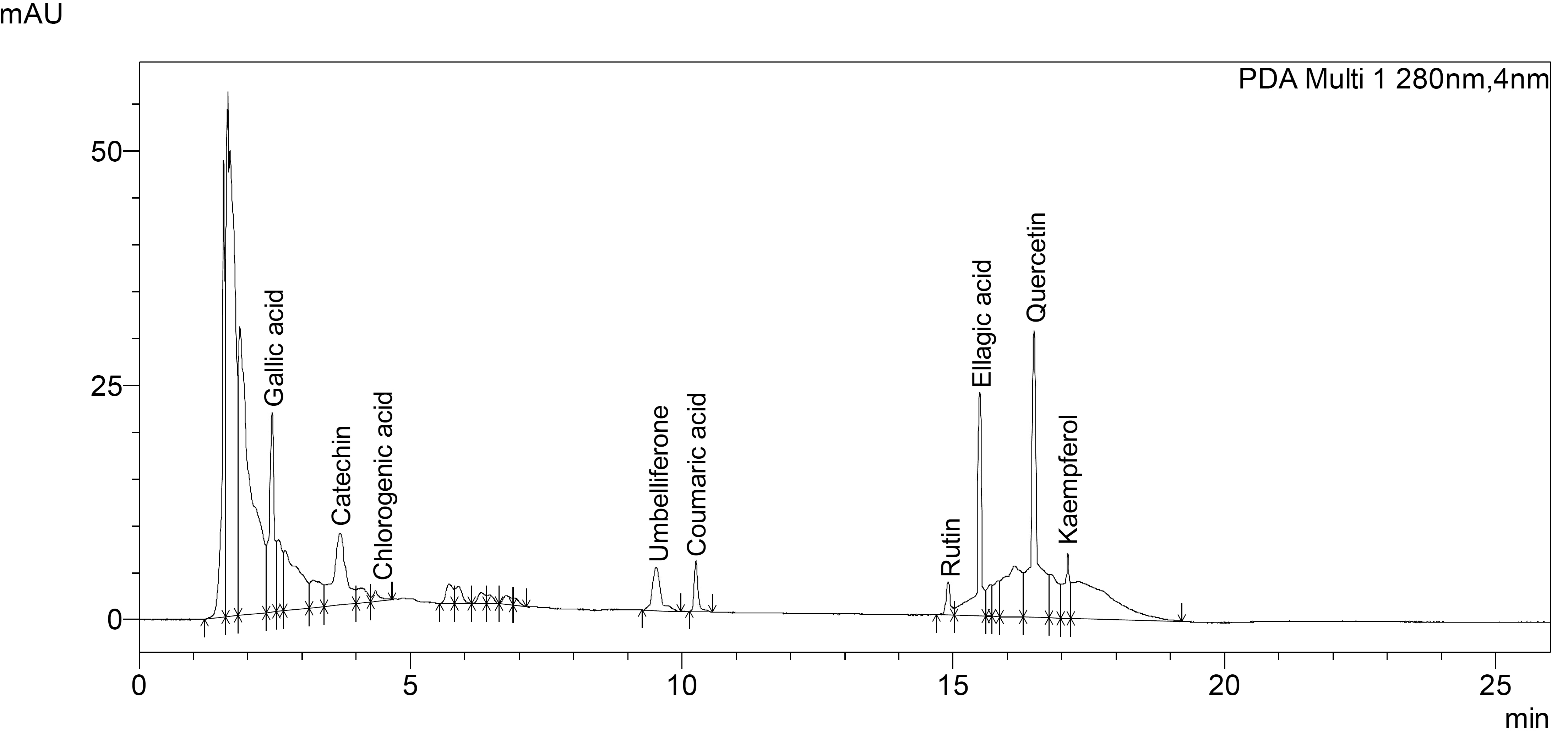


**Figure S6.** HPLC-PDA chromatogram of *Trigonella corniculata* L. (seed) extract

**Figure S7.** Percent decrease in Rhodamine intensity in HepG2 cells after treatment with IC_50_ value of Herbal combination
